# Altered Amygdala Connectivity in Individuals with Chronic Traumatic Brain Injury and Comorbid Depressive Symptoms

**DOI:** 10.3389/fneur.2015.00231

**Published:** 2015-11-04

**Authors:** Kihwan Han, Sandra B. Chapman, Daniel C. Krawczyk

**Affiliations:** ^1^Center for BrainHealth^®^, School of Behavioral and Brain Sciences, University of Texas at Dallas, Dallas, TX, USA; ^2^Department of Psychiatry, University of Texas Southwestern Medical Center, Dallas, TX, USA

**Keywords:** TBI, depression, functional connectivity, fMRI, amygdala, resting-state, BDI, beck depression inventory-II

## Abstract

Depression is one of the most common psychiatric conditions in individuals with chronic traumatic brain injury (TBI). Though depression has detrimental effects in TBI and network dysfunction is a “hallmark” of TBI and depression, there have not been any prior investigations of connectivity-based neuroimaging biomarkers for comorbid depression in TBI. We utilized resting-state functional magnetic resonance imaging to identify altered amygdala connectivity in individuals with chronic TBI (8 years post-injury on average) exhibiting comorbid depressive symptoms (*N* = 31), relative to chronic TBI individuals having minimal depressive symptoms (*N* = 23). Connectivity analysis of these participant sub-groups revealed that the TBI-plus-depressive symptoms group showed relative increases in amygdala connectivity primarily in the regions that are part of the salience, somatomotor, dorsal attention, and visual networks (*p*_voxel_ < 0.01, *p*_cluster_ < 0.025). Relative increases in amygdala connectivity in the TBI-plus-depressive symptoms group were also observed within areas of the limbic–cortical mood-regulating circuit (the left dorsomedial and right dorsolateral prefrontal cortices and thalamus) and the brainstem. Further analysis revealed that spatially dissociable patterns of correlation between amygdala connectivity and symptom severity according to subtypes (Cognitive and Affective) of depressive symptoms (*p*_voxel_ < 0.01, *p*_cluster_ < 0.025). Taken together, these results suggest that amygdala connectivity may be a potentially effective neuroimaging biomarker for comorbid depressive symptoms in chronic TBI.

## Introduction

Depression is one of the most common psychiatric conditions among individuals with traumatic brain injury (TBI) (1–5). For example, a recent study with large sample size (*N* = 559) showed that 53.1% met criteria for major depressive disorder during the first year following the occurrence of a TBI (6). The comorbidity of depression in TBI is associated with poorer cognitive function (7–9), reduced health-related quality of life (6, 10, 11), greater functional disability (3, 12), increased suicide attempts (13), greater sexual dysfunction (14), less social and recreational activity (15), and poorer recovery (16). Given the significant effects of comorbid depression on individuals with TBI, it is important to better understand the underlying neural mechanisms of depression in the context of TBI.

Structural and functional connectivity utilizing advanced neuroimaging techniques, such as diffusion tensor imaging (DTI) and functional magnetic resonance imaging (fMRI), revealed that network dysfunction is a hallmark of TBI, particularly mild TBI [see Sharp et al. (17) for a review]. For example, resting-state functional connectivity MRI has been used to identify alterations in the default mode network (DMN) (18, 19), executive network (19), inter-hemispheric connectivity (20), thalamic connectivity (21), small-worldness (22, 23), and modular organization (24) of individuals with TBI. The primary injury mechanism responsible for such network dysfunction following TBI is diffuse axonal injury (DAI) (25). Since DAI occurs at multiple white matter pathways connecting distributed regions across the brain, the effects of DAI are complex. Furthermore, DAI may interact with pathology associated with complications arising late in life after TBI such as Alzheimer’s disease or chronic traumatic encephalopathy (17). Thus, connectivity-based assessments of individuals with TBI may continue to provide valuable information for us to better understand the complex nature of clinical outcomes following TBI.

Depression is currently viewed as a system-level disorder that affects (1) integrated pathways linking limbic, cortical, and subcortical brain regions (26) and (2) neurotransmitter activity related to these regions (27). Since depression is fundamentally a mood disorder, investigating neural processes occurring over extended periods of time (minutes or hours) rather than brief periods of time (such as individual trials lasting only seconds in a task-based fMRI session) may be more relevant to our understanding of depression (28). Thus, the research community has increasingly relied upon resting-state fMRI (rsfMRI) to investigate neural function in individuals diagnosed with depression [see Dutta et al. (29), Hamilton et al. (28), Northoff et al. (30), and Wang et al. (31) for a review]. For example, previous resting-state fMRI studies of depression have revealed increased DMN connectivity in the subgenual anterior cingulate cortex and thalamus (32), reduced frontoparietal control network (FPCN) (33) connectivity (34), reduced salience network (SN) (35) connectivity involving the anterior insula (36), and elevated connectivity of the dorsomedial prefrontal cortex (DMPFC) to the DMN, cognitive control, and affective networks (ANs) (37) in individuals with depression. These reductions and increases in connectivity related to depression provide a complex picture at the present time. The directionality of altered connectivity may depend upon a variety of factors, including brain regions involved, age, and comorbid conditions.

Given the central role of the amygdala in both bottom-up and top-down emotional processes (38), the amygdala and its connecting regions have been widely studied in the depression literature (39–48). From a network perspective, the amygdala has connections with the cortical–striatal–pallidal–thalamic circuit, which is often considered to be the core neural system in mood disorders (49). More specifically, the amygdala connects (1) the medial prefrontal cortex, a part of the DMN (50), and (2) anterior insula and hypothalamus, both of which are parts of the SN (35). Since alterations in the DMN and SN of individuals TBI have been previously reported (18, 19, 51), it is important to investigate whether amygdala connectivity can be a potential neuroimaging biomarker for comorbid depression among individuals at the chronic phase of a TBI.

Though two separate lines of research in (1) TBI without depression and (2) depression without TBI have both demonstrated marked network dysfunction of those individuals, there have been no functional or structural connectivity studies in individuals with *both* TBI and depression. As such, most of the studies of neuroimaging biomarkers for comorbid depression among individuals with TBI have been limited to regional assessments of brain structure and function, including regional gray matter volume (8, 52–55), white mater integrity (56–58), presence of microbleeds (31), and regional brain activity (40, 41, 59–61). Thus, connectivity-based studies in TBI with comorbid depressive symptoms can contribute to this body of literature and are well suited to capturing the larger-scale network interactions associated with depressive symptoms in this population.

Depression is a psychological construct comprised of several factors (62). Previous investigations of the neural correlates of depression severity in TBI (41, 52, 54, 56) did not specifically characterize the neural correlates in accordance with subtypes of depressive symptoms [see Strain et al. (58) for an exception]. Given the heterogeneity of both depressive symptoms and TBI, several studies attempted to identify the underlying factor structure of depressive symptoms among TBI individuals (63–65). Thus, identifying neural correlates of these underlying factors will be useful for assessment, diagnosis, and characterization of often heterogeneous TBI population (66).

Here, we utilized resting-state fMRI to identify altered amygdala connectivity within individuals with chronic TBI and comorbid depressive symptoms. Based on previous findings reporting abnormal amygdala connectivity in depression and aberrant connectivity in TBI, we hypothesized that the amygdala connectivity of TBI individuals with comorbid depressive symptoms would be altered, relative to TBI individuals exhibiting minimal depressive symptoms. We also predicted that amygdala connectivity would be characterized by abnormal spatial patterns associated with different subtypes of depressive symptoms according to the Buckley categories of items from the Beck Depression Inventory (BDI), which classifies depression into separable constructs representing cognitive, somatic, and emotional symptoms (62, 67).

## Materials and Methods

### Participants

The data used for this analysis are part of an ongoing study (68). We analyzed 54 chronic TBI individuals who ranged from lower moderate disability to lower good recovery [age 20–60; >6 months post-injury; 5–7 on the extended Glasgow outcome scale (GOS-E)] (69), who completed MRI scans and whose MRI scans passed quality assurance (QA) procedures described below. We recruited these participants from the Dallas–Ft. Worth community and screened by a phone interview before inclusion in the study. The primary causes of TBIs are blasts, blunt force trauma, falls, athletic impacts, vehicle accidents, or combinations thereof. Note that, at several years post-injury time, it was not feasible to obtain all participants’ clinical information on initial injury characteristics such as Glasgow coma scale (GCS) (70) or the duration of loss of consciousness (LOC) from the inpatient, acute-care facilities where they were hospitalized multiple years ago (see the limitation section regarding limited clinical information on initial injury). Therefore, injury severity and the duration of LOC at the time of injury were estimated utilizing the Ohio State University TBI identification (OSU TBI-ID) method (71). The OSU TBI-ID method is a structured interview developed to incorporate recommendations from the Centers for Disease Control and Prevention (CDC) for the detection of TBI history. The OSU TBI-ID first elicits recall of all possible head or neck injuries receiving medical attention, or that should have received medical attention. The elicitation method subsequently focuses on any injuries involving a blow to the head or neck, fall, blast exposure, or vehicle accident that can cause an injury to the brain. For these injuries, the occurrence and nature of altered consciousness and treatment received are probed. Using the OSU TBI-ID, various pieces of information on injury history are available, including injury severity, the number of injuries, worst injury, age at injury, and time since the most recent injury. Bogner and Corrigan (72) and Corrigan and Bogner (71) showed that the OSU TBI-ID method had good inter-rater reliability and test–retest reliability. Previous studies (71, 72) demonstrated the validity of summary indices driven from the OSU TBI-ID to predict TBI-related cognitive and behavioral deficits. Note that the OSU TBI-ID method estimates initial injury severity based on the duration of LOC and the CDC guidelines for the conceptual definition and identification of TBI (73, 74). Specifically, the TBI participants whose duration of LOC <30 min, <24 h, or >24 h were considered to be *probable* mild, *probable* moderate, or *probable* severe TBI, respectively. The participants included both civilians and veterans (see Table 1 for demographics). No participants had a history of any significant, clinically diagnosed neurological or psychiatric comorbidities. Participants also had no history of depressive symptoms prior to their TBI or TBIs. We also confirmed that all participants’ brains were free of visible focal lesions, contusions, mass shifting, or extreme cortical thinning on structural MRI scans. This confirmation should rule out potential effects of such macro structural injuries on fMRI preprocessing steps, including registration and subsequent functional connectivity analyses. All participants provided written informed consent. This study was conducted in compliance with the declaration of Helsinki. The study was approved by the Institutional Review Boards of the University of Texas at Dallas and University of Texas Southwestern Medical Center.

**Table 1:** **Demographics**.

Demographics	TBI-plus-depressive symptoms^a^	TBI-only^b^	Stat	DF	*p*-value^c^	CI	ES
Number of participants	31	23	–	–	–	–	–
Age (years)^d^	38.8 ± 11.2	39.0 ± 11.7	−0.1	46.3	0.96	(−6.5, 6.2)	−0.01
Education (years)^d^	15.9 ± 2.9	15.7 ± 1.8	0.3	51.1	0.77	(−1.1, 1.5)	0.08
Gender (males, females)	19, 12	14, 9	1.0	–	1.00	(0.3, 3.0)	0.98
Civilians, veterans	19, 12	16, 7	1.4	–	0.58	(0.5, 4.5)	1.44
Post-injury time (years)^d^	8.9 ± 10.0	7.6 ± 6.3	883	–	0.74	(−3.4, 2.3)	0.05
Estimated injury severity (mild, moderate, severe)^e^	21, 4, 6	17, 3, 3	0.4	2	0.82	–	0.08
Primary cause of injury (blast, blunt force trauma, fall, athletic impacts, vehicle accidents, combined)	3, 4, 6, 3, 8, 7	2, 4, 2, 3, 5, 7	1.7	5	0.88	–	0.18
Estimated LOC (<30 min, <1 day, >1 day)	21, 4, 6	17, 3, 3	0.4	2	0.82	–	0.08
PCL-S^d^	50.1 ± 15.4	31.7 ± 12.5	5.0	51.5	****<**10**^−^**^5^**	(11.6, 26.9)	1.33
BDI-II total^d^	22.4 ± 6.4	7.3 ± 4.1	–	–	–	–	–
BDI-II Buckley cognitive^d^	8.3 ± 3.5	1.6 ± 1.6	9.4	44.4	****<**10**^−^**^11^**	(5.2, 8.1)	2.30
BDI-II Buckley affective^d^	4.7 ± 2.2	1.9 ± 1.6	5.5	52.0	****<**10**^−^**^5^**	(1.8, 3.9)	1.42
BDI-II Buckley somatic^d^	9.4 ± 3.8	3.7 ± 2.4	6.7	51.2	****<**10**^−^**^7^**	(4.0, 7.4)	1.72
Motion-censored volumes (%)^d^	16.6 ± 13.5	12.0 ± 8.5	911	–	0.31	(−2.5, 7.7)	−0.14
FD after censoring and trimming (mm)^d^	0.17 ± 0.05	0.15 ± 0.04	1.6	49.1	0.11	(<−0.1, <0.1)	0.43

*LOC, loss of consciousness; PCL-S, Post-traumatic Stress Disorder Check List Stressor-specific; BDI-II, Beck Depression Inventory-II; FD, framewise displacement; Stat, statistical value; DF, degrees of freedom; CI, confidence interval; ES, effect size*.

*^a^BDI-II of 14–63*.

*^b^BDI-II of 0–13*.

*^c^Bold face indicates *p* < 0.05*.

*^d^Mean and SD values were reported*.

*^e^Based on the OSU TBI screening form (71)*.

### Assessment of Depressive Symptoms

Depressive symptom severity was quantified using the Beck Depression Inventory-II (BDI-II) (75). In the development of the BDI test, Beck et al. (75) showed that the BDI-II had excellent internal consistency for the 500 psychiatric outpatients (α = 0.92) and the 120 college students (α = 0.93) and a robust 1-week test–retest correlation (*r* = 0.93). They also reported highly convergent and discriminant validity of the BDI-II with respect to clinically rated depression and anxiety such as the Revised Hamilton Psychiatric Rating Scale for Depression (76) and the Revised Hamilton Anxiety Rating Scale (77). According to suggested total BDI score guidelines for the diagnosis of major depression (75), we subdivided the TBI participants into two groups: a TBI with minimal depressive symptom group (*N* = 23; 0–13) and a TBI with mild to severe depressive symptom group (*N* = 31; 14–63). Due to a previous report detailing altered amygdala connectivity in post-traumatic stress disorder (PTSD) (78, 79), we also measured PTSD symptom severity of the TBI participants enrolled in this study using the PTSD Check List Stressor-specific (PCL-S) (80) for the forth edition of the American Psychiatric Association’s Diagnostic and Statistical Manual of Mental Disorders (DSM-IV).

### Neuropsychological Assessments

We administered neuropsychological tests on the participants to characterize the TBI sub-groups in a variety of domains. These tests include similarities, matrix reasoning, and full scale intelligent quotient-2 (FSIQ-2) from the Wechsler Abbreviated Scale of Intelligence (WASI) for estimated current IQ (81), FSIQ from the Wechsler Test of Adult Reading (WTAR) for estimated premorbid IQ (82), digit span forward and backward from the Wechsler Adult Intelligence Scale-Third Edition (WAIS-III) for working memory (83), color-word, verbal fluency, card sorting, trail making from the Delis–Kaplan Executive Function System (D–KEFS) for inhibitory control, switching, verbal fluency, processing speed and problem solving (84), immediate recall and delayed recall from the Wechsler Memory Scale-Fourth Edition (WMS-IV) for memory and recall (85), verbal problem solving assessment (S. B. Chapman, unpublished data), and visual selective learning task adapted from Hanten et al. (86). We also assessed satisfaction with life scale (87) for the participants to measure global cognitive judgments of their life satisfaction.

### MRI Data Acquisition

The participants underwent MRI scanning on a Philips Achieva 3 T scanner (Philips Medical Systems, Netherlands) at the Advanced Imaging Research Center at the University of Texas Southwestern Medical Center. In each imaging session, one or two 416-s runs of rsfMRI were acquired using a standard 32-channel head coil with T_2_*-weighted image sequence (repetition time (TR)/echo time (TE) = 2000/30 ms; flip angle (FA) = 80°; field of view (FOV) = 22.0 cm × 22.0 cm; matrix = 64 × 64; 37 slices, 4.0 mm thick). During rsfMRI acquisition, the participants were asked to remain still with their eyes closed. For rsfMRI alignment, we obtained one high-resolution T_1_-weighted image of the whole brain (TR/TE = 8.2/3.8 ms; FA = 12°; FOV = 25.6 cm × 25.6 cm; matrix = 256 × 256; 160 slices, 1.0 mm thick) for each participant using the same head coil.

### MRI Preprocessing

Resting-state fMRI data were preprocessed with standard methods using a modified version of a shell script generated by http://afni_proc.py^1^ from AFNI (88). Each subject’s whole-brain structural images were first skull-stripped and registered (affine transform with 12 parameters) to the Montreal Neurological Institute (MNI) space (89). For each rsfMRI run, the initial four time points were discarded to allow T_1_ magnetization saturation. Standard preprocessing methods were then applied, including despiking, slice timing correction, motion correction, coregistration to the structural images in the MNI space using a single affine transform with spatial resampling (4 mm isotropic), normalization to whole-brain mode of 1000, band-pass filtering (0.009 < *f* < 0.08 Hz), and linear regression. At the motion correction stage, the six rigid body motion profiles were obtained for the linear regression. In the linear regression, the rsfMRI time series were third order detrended, and several sources of signal fluctuation unlikely to be of neuronal origin were regressed out as nuisance variables: (1) six parameters for the rigid body head motion acquired from the motion correction (90), (2) the signal averaged over the lateral ventricles (91), (3) the signal averaged over a region centered in the deep cerebral white matter (91), and (4) the first temporal derivatives of aforementioned parameters. Note that we did not apply global signal regression in this procedure since global signal regression could arguably generate difficulties in interpreting group comparisons (92–96). After the linear regression, motion “scrubbing” (97) was performed with a framewise displacement (FD) of 0.5 mm and a standardized DVARS^2^ of 1.8 to prevent potential motion artifacts (97–99). A standardized DVARS of 1.8 corresponds to the median plus 1.5 times interquartile range of the standardized DVARS data across all frames and runs. The remaining rsfMRI signals were spatially blurred with 6-mm full-width-at-half-maximum (FWHM) Gaussian kernel. For participants on whom two runs of rsfMRI scans were acquired, the two preprocessed rsfMRI runs were temporally concatenated. To account for the differences in total number of frames (subsequently different degrees of freedom for correlation coefficients) after motion scrubbing across rsfMRI scans, all remaining frames were trimmed to the minimum length (121 frames; 242 s) across all rsfMRI scans as suggested in Power et al. (100).

### Quality Assurance

We visually inspected all structural MRI scans to ensure that subjects had no significant brain atrophy. In rsfMRI preprocessing, the quality of the preprocessed data was visually inspected at each step. After motion “scrubbing,” we confirmed that the total time duration of remaining frames after the “scrubbing” exceeded 4 min, the minimum length required to reliably estimate functional connectivity (101).

### Whole-Brain Seed-Based Connectivity Analysis

For each participant, amygdala connectivity was identified by seeding the center of mass (L: −24, −1, −16; R: 26, 1, −18) of the amygdala labels in the automated anatomical label (AAL) atlas (102) with 5-mm radius spheres, respectively. Pearson correlation maps were then Fisher’s *Z*-transformed to ensure the normality of correlations, followed by conversions to *z*-scores (i.e., normal distributions with zero mean and unit variance). To identify patterns of group differences in amygdala connectivity, we performed the general linear model (GLM) analysis at each of the voxels with regressors of group memberships, age, PCL-S scores, age by PCL-S score interactions, and FD after censoring and trimming. Specifically, connectivity strength for the participants at each of the voxels was modeled as follows:

$$\begin{array}{c} y=\beta_{1}+\beta_{2}\cdot I_{\text{D}}+\beta_{3}\cdot a\text{+}\beta_{4}\cdot I_{\text{C}}\cdot p+\beta_{5}\cdot I_{\text{D}}\cdot p\;+\beta_{6}\cdot I_{\text{C}}\cdot a\cdot p+\beta_{7}\cdot I_{\text{D}}\cdot a\cdot p+\beta_{8}\cdot f+e \end{array}$$

where vectors *a*, *p*, and *f* are age, PCL-S scores, and FD after censoring and trimming for the participants, *I_C_* and *I_D_* are group indicator vectors for the controls and depressive symptoms group, and *e* is a normally distributed vector with zero mean, respectively. The age values were included in this model because of reports of significant age effects on amygdala connectivity in healthy individuals (103), and the PCL-S scores were included to account for potential effects of PTSD on amygdala connectivity (78, 79). FD after censoring and trimming was included to account for potential effects of trends in higher FD in TBI-plus-depressive symptoms group. To construct covariate regressors, the age and FD values were centered at the global mean over the two groups after confirming no statistically significant group differences whereas the PCL-S scores were within-group centered to interpret the between-group differences in amygdala connectivity at their respective average PCL-S scores. The reason for applying within-group centering to the PCL-S scores was due to reported comorbidities between depression and PTSD in TBI (104) and statistically significant group differences in the PCL-S scores for our groups (see Table 1 and limitations relevant to comorbidity between depression and PTSD in TBI). Statistically significant group differences in amygdala connectivity at the whole-brain level were identified at *p*_voxel_ < 0.01, correcting for multiple comparisons across voxels by cluster size using AFNI’s AlphaSim and across the number of amygdala seeds by additional Bonferroni correction at *p*_cluster_ < 0.05/2 (20 voxels; 1,280 mm^3^).

### Conjunction Analysis

To identify regions showing consistent group differences in both left and right amygdala connectivity, we performed a conjunction analysis. This was performed in accordance with the conjunction inference procedure described by Nichols et al. (105). We took minimum statistics over the group comparison maps for left and right amygdala connectivity at each of the voxels then thresholded the minimum statistic map over the whole brain at *p*_voxel_ < 0.01 and *p*_cluster_ < 0.025. We identified peak foci of the conjunction map as follows. First, we generated local peaks within the given cluster with >8 mm apart. Next, we selected a local peak closest to the center of mass for the cluster as a focus of the cluster. To elucidate spatial patterns of altered amygdala connectivity at the large-scale network level, we also overlaid the conjunction map onto network-based parcelation maps of the cerebral cortex and cerebellum (106, 107).

### Correlation Analysis

We performed a correlation analysis to identify spatial patterns of amygdala connectivity associated with sub-factors of depressive symptoms within the TBI-plus-depressive symptoms group. We first obtained participants’ BDI-II sub-factor scores using the Buckley three-factor model (67). The Buckley three-factor model decomposes the total BDI-II scores into cognitive, affective, and somatic symptoms (see Table 2). The cognitive factor includes items regarding sadness, pessimism, past failure, guilty feelings, punishment feelings, self-dislike, self-criticalness, suicidal ideation, and worthlessness. The affective factor includes items probing loss of pleasure, crying, loss of interest, and indecisiveness. The somatic factor includes the agitation, loss of energy, sleep disturbance, irritability, appetite disturbance, concentration difficulty, fatigue, and loss of sexual interest test items. The Buckley factor model was originally proposed for treatment-seeking substance abuser (67). The Buckley factor model has been reported to provide a better characterization of depressive symptom severity of psychiatric patients over alternative models (108) and served as the best model for veterans with polytrauma (63). Regions with statistically significant correlation between amygdala connectivity and each of the Buckley BDI-II sub-scores were then identified at *p*_voxel_ < 0.01, correcting for multiple comparisons by cluster size using AFNI’s AlphaSim and the number of amygdala seeds (Bonferroni) at *p*_cluster_ < 0.05/2 (20 voxels; 1,280 mm^3^).

**Table 2:** **Buckley BDI-II factor structure**.

#	Factor^a^	Test item
1	Cognitive	(1) Sadness
2	Cognitive	(2) Pessimism
3	Cognitive	(3) Past failure
4	Cognitive	(5) Guilty feelings
5	Cognitive	(6) Punishment feelings
6	Cognitive	(7) Self-dislike
7	Cognitive	(8) Self-criticalness
8	Cognitive	(9) Suicidal thoughts or wishes
9	Cognitive	(14) Worthlessness
10	Affective	(4) Loss of pleasure
11	Affective	(10) Crying
12	Affective	(12) Loss of interest
13	Affective	(13) Indecisiveness
14	Somatic	(11) Agitation
15	Somatic	(15) Loss of energy
16	Somatic	(16) Changes in sleeping pattern
17	Somatic	(17) Irritability
18	Somatic	(18) Changes in appetite
19	Somatic	(19) Concentration difficulty
20	Somatic	(20) Tiredness or fatigue
21	Somatic	(21) Loss of interest in sex

*^a^The Buckley three-factor model (67)*.

### Data-Driven Connectivity Analysis Over 268 Putative Functional Nodes

To identify (1) if the amygdala is the most important driver of observed group differences in connectivity strength and the patterns of correlation with the Buckley BDI factor scores or, alternatively, (2) if these effects can be better represented via other components of the distributed network, we assessed group differences in connectivity strength and correlation coefficients over 268 putative functional nodes. As in Cao et al. (109), the 268 nodes were obtained by combining 264 nodes reported by Power et al. (110), the hippocampi (L: −30, −13, −12; R: 30, −4, −12) from Bilek et al. (111) and the amygdalae. For the defined regions, we constructed a Fisher’s *Z*-transformed connectivity matrix followed by a *Z*-score (zero mean and unit variance) conversion. For group comparisons, we performed the GLM analysis at each pair of the normalized (*Z*-scored) connectivity matrix elements. Subsequently, we obtained average group differences in connectivity strength for a node (seed) by taking average absolute values of *Z*-statistics for connections between the given node and the other 267 nodes. For correlations between connectivity and the Buckley BDI factor scores, we obtained Fisher’s *Z*-transformed and normalized (*Z*-scored) correlation coefficients for each pair of nodes. Subsequently, we calculated average correlations between BDI factors scores and connectivity strength with a node by taking average absolutes values of normalized correlation coefficients for connections between the given node and the other 267 nodes.

### Statistical Analyses

All statistical analyses were carried out in MATLAB R2013a. First, we performed the Shapiro–Wilk test at α = 0.05 to assess the normality of age, years of education, post-injury time, PCL-S total scores, BDI-II total scores, Buckley BDI factor scores, percentage of motion-censored volumes, and average FD after motion censoring and trimming within each of the groups. Post-injury time and percentage of motion-censored volumes did not pass Shapiro–Wilk normality test. Thus, the Wilcoxon rank-sum test was used to compare these measures between the groups. Two sample *t*-tests were used to compare age, years of education, PCL-S total scores, BDI-II total, and Buckley BDI-II factor scores between the groups. The Fisher’s exact test was used to compare the gender distributions and proportion of civilians and veterans between the groups. The likelihood ratio chi-square test was used to compare the distribution of primary cause of injury between the groups. We performed linear regression analysis on neuropsychological measures with age and education covariates.

In these statistical tests, 95% confidence intervals were also obtained as follows: mean of group differences for the *t*-test, median of group differences for the Wilcoxon rank-sum test, odds ratio for the Fisher’s exact test, and group contrasts for the regression analysis. Effect sizes were also obtained for each type of test: Hedge’s g for the *t*-test, W for the first group for the Wilcoxon rank-sum test, odds ratio for the Fisher’s exact test, Cramer’s V for the chi-square test, and η_p_^2^ for the linear regression analysis. To identify if group differences in amygdala connectivity in the GLM analysis have sufficient power, we obtained observed power at each of the voxels and thresholded at 0.8.

### Control Analyses

#### Somatomotor Connectivity

To ensure data quality of resting-state functional connectivity of the TBI sub-groups, we obtained the somatomotor connectivity by seeding at somatomotor cortices (L: −41, −18, 59; R: 46, −19, 54) with a 5-mm radius sphere. The seed locations were obtained after converting Talairach coordinates reported by Fox et al. (112) to MNI coordinates using “tal2icbm” program^3^ (113).

#### Group Comparisons with Healthy Individuals

To confirm that the TBI participants had residual TBI-related deficits in neuropsychological behavior, we compared neuropsychological assessment results of each of the TBI sub-groups with those of 17 healthy individuals whose MRI scans were acquired from the same MRI scanner and same imaging parameters as those of the TBI sub-groups (see Table S1 in Supplementary Material for demographics of the healthy individuals). In addition, we obtained connectivity in the three groups by seeding the posterior cingulate cortex [PCC; L: −7, −55, 27; R: 8, −48, 31; Power et al. (110)] and anterior prefrontal cortex [aPFC; L: −36, 57, 9; R: 34, 52, 10; Vincent et al. (33)] with a 5-mm radius sphere, respectively, which are parts of the DMN (50) and FPCN (33). We expected that DMN and FPCN would be likely to show alterations in the TBI sub-groups relative to the healthy group (18, 19, 114). Furthermore, to observe amygdala connectivity of the TBI sub-groups in the context of healthy individuals, we performed the GLM analysis with the three groups without PCL-S score covariates. Note that, in these group comparisons of the neuropsychological test performance and connectivity measures, we excluded older TBI participants to match their age with the healthy individuals at α = 0.05 as there were statistically significant group differences in age with the full TBI samples at α = 0.05 (see Table S1 in Supplementary Material for the sample sizes of the age-matched TBI sub-groups). We further confirmed that there were no statistically significant effects of age on any of the neuropsychological assessment scores or any of the connectivity measures for the age-matched TBI sub-groups and the healthy group even though there were still trends that the age-matched TBI sub-groups were older than the healthy group (see Table S1 in Supplementary Material).

#### Assessment of Amygdala Connectivity in Civilians Versus Veterans Within the TBI-Plus-Depressive Symptoms Group

To identify if mixed veterans and civilians within the TBI-plus-depressive symptoms group systematically affected the correlation analysis results, we further subdivided the TBI-plus-depressive symptoms group into civilians and veterans, then we compared their respective amygdala connectivity at peak locations within the regions showing statistically significant correlations between amygdala connectivity and the BDI sub-scores.

#### Assessment of the Effects of Comorbid PTSD Symptom Severity on Amygdala Connectivity

To assess the effects of comorbid PTSD symptom severity on group comparison results for amygdala connectivity in the GLM analysis, we also obtained color maps for these covariates at *p*_voxel_ < 0.01 and *p*_cluster_ < 0.025.

#### Assessment of the Effects of Estimated Injury Severity on Depressive Symptom Severity and Amygdala Connectivity

To identify if there were systematic effects of estimated injury severity on our findings, we assessed the BDI-II total scores and amygdala connectivity of the TBI sub-groups according to estimated injury severity, and we repeated group analyses of amygdala connectivity with *probable* mild TBI participants only (*N* = 21 for the TBI-plus-depressive symptoms group and *N* = 17 for the TBI-only group). Furthermore, we performed group analyses of amygdala connectivity by excluding *probable* mild TBI participants while matching the sample sizes for each of the newly formed TBI sub-groups with those of the sub-groups of participants with mild TBI only. The goal of this analysis was to test whether the *probable* moderate and severe participants contributed more to the amygdala connectivity differences than the *probable* mild TBI participants. To achieve this goal, we utilized a resampling method, conceptually similar to the resampling procedure described in Han and Talavage (115). Specifically, we resampled the TBI sub-groups by excluding 10 (out of 21) and 6 (out of 17) *probable* mild TBI participants from the original TBI-plus-depressive symptoms and TBI-only groups, respectively, in a pseudo-random fashion. Then, we performed group analyses of amygdala connectivity in the resampled TBI sub-groups. We repeated this resampling procedure 5,000 times. Subsequently, we defined the magnitude of overall group differences in amygdala connectivity by taking average absolute values of *Z*-statistics for the group comparison test over the whole brain. Finally, to determine if the magnitude of overall group differences in amygdala connectivity of the *probable* mild TBI participants only groups was “significantly” different than those of the resampled TBI sub-group (i.e., higher proportions of *probable* moderate and severe TBI participants than the original TBI groups), we assessed whether the magnitude of overall group differences in amygdala connectivity of the *probable* mild TBI sub-groups fell outside the intervals of 2.5–97.5th percentile (similar to 95% confidence intervals for *Z*-statistics) for group differences, obtained from 5,000 resampled groups.

### Visualization

The thresholded volumetric statistical results for group differences in amygdala connectivity and correlation analysis were surface-projected onto the cortical surface of the population-averaged landmark- and surface-based (PALS-B12) atlas (116) using a multi-fiducial mapping that avoids the biases of choosing a cortical surface from a single-individual as an atlas target, implemented in Caret Software (117).

## Results

### Demographics

The TBI participants were in the long-term chronic phase of TBI (approximately 8 years post-injury time on average). There were no statistically significant group differences in age, education, gender, proportion of civilians and veterans, post-injury time, distributions of estimated injury severity or primary injury types, percentage of motion-censored volumes, or gender (Table 1). The TBI-plus-depressive symptoms group had statistically significant higher scores on the PCL-S and Buckley BDI factors over the TBI-only group. The finding of higher PTSD symptom severity in the TBI-plus-depressive symptoms group is consistent with findings reported by Hibbard et al. (4) and Levin et al. (104). We also observed a trend in which the TBI-plus-depressive symptoms group had higher FD after censoring and trimming than the TBI-only group. Thus, FD after censoring and trimming was included as a covariate in all subsequent analyses.

### Neuropsychological Measures

There were no statistically significant group differences in estimated premorbid IQ, but group differences in current IQ showed marginal statistical significance (Table 3). The TBI-plus-depressive symptoms group showed poorer performance in immediate and delayed recall, consistent with previously reported on memory deficits among depressive TBI individuals (9, 60). Group analyses of neuropsychological measures also revealed group differences in category fluency scores of the verbal fluency test. Relatively poor performance in these neuropsychological measures of the TBI-plus-depressive symptoms group indicates that there were adverse effects of depressive symptoms likely influencing cognitive function among individuals with chronic TBI. We also observed relatively lower satisfaction with life scale in the TBI-plus-depressive symptoms group, suggesting the presence of adverse effects of depressive symptoms on global cognitive judgments of life satisfaction among individuals with chronic TBI.

**Table 3:** **Neuropsychological assessment results**.

Neuropsychological measure^a^	TBI-plus-depressive symptoms	TBI-only	*T*	DF	*p*-value^b^	CI	**η**_p_^2^
Similarities	37.7 ± 3.9	38.5 ± 3.7	0.4	50	0.41	(−3.0, 1.2)	0.01
Matrix reasoning	27.7 ± 4.2	28.5 ± 3.9	−0.8	50	0.43	(−3.0, 1.3)	0.01
WASI FSIQ-2 (current IQ)	108.6 ± 10.3	113.7 ± 9.7	−2.1	50	**0.04**	(−11.0, −0.2)	0.08
WTAR FSIQ (premorbid IQ)	109.4 ± 8.2	111.1 ± 7.9	−1.1	50	0.28	(−6.4, 1.9)	0.02
Digit span forward	10.6 ± 2.1	10.8 ± 2.2	−0.3	50	0.75	(−1.4, 1.0)	<0.01
Digit span backward	7.0 ± 2.2	7.5 ± 2.1	−0.9	50	0.36	(−1.7, 0.6)	0.02
Color-word: color naming (s)	30.4 ± 7.7	29.3 ± 5.0	0.6	50	0.55	(−2.6, 4.9)	0.01
Color-word: word reading (s)	24.3 ± 6.9	22.3 ± 5.2	1.3	50	0.20	(−1.2, 5.7)	0.03
Color-word: inhibition (s)	59.0 ± 14.6	53.6 ± 11.5	1.6	50	0.11	(−1.4, 13.0)	0.05
Color-word: inhibition/switching (s)	67.3 ± 15.7	60.0 ± 13.7	2.0	50	0.06	(−0.2, 16.0)	0.07
Verbal fluency: letter fluency, total correct	39.9 ± 9.1	42.1 ± 10.6	−0.9	50	0.35	(−7.9, 2.9)	0.02
Verbal fluency: category fluency, total correct	38.9 ± 8.3	46.4 ± 8.3	−3.3	50	**0.002**	(−12.3, −2.9)	0.18
Verbal fluency: category switching, total correct	14.7 ± 2.6	14.7 ± 2.9	−0.0	50	0.98	(−1.5, 1.5)	<0.01
Verbal fluency: category switching, total switching accuracy	13.7 ± 2.8	13.9 ± 2.8	−0.2	50	0.81	(−1.7, 1.3)	<0.01
Sorting: free sorting, confirmed correct sorts	9.3 ± 2.3	9.7 ± 2.6	−0.8	50	0.41	(−1.7, 0.7)	0.01
Sorting: free sorting, description score	35.7 ± 9.7	37.4 ± 10.5	−0.9	50	0.39	(−7.6, 3.0)	0.02
Sorting: sort recognition, description score	36.8 ± 11.1	34.7 ± 12.5	0.6	50	0.58	(−4.7, 8.3)	0.01
Sorting: combined description score	72.5 ± 19.2	72.1 ± 21.4	−0.1	50	0.93	(−11.5, 10.5)	<0.01
Trail making: visual scanning (s)	18.9 ± 4.7	17.7 ± 5.6	1.0	50	0.33	(−1.4, 4.2)	0.02
Trail making: number sequencing (s)	28.5 ± 8.7	28.0 ± 10.2	0.2	50	0.84	(−4.6, 5.7)	<0.01
Trail making: letter switching (s)	27.9 ± 7.6	25.6 ± 8.2	1.1	50	0.30	(−2.1, 6.7)	0.02
Trail making: number-letter switching (s)	72.9 ± 26.0	65.0 ± 20.2	1.3	50	0.19	(−4.5, 21.8)	0.04
Trail making: motor speed (s)	21.1 ± 8.3	20.1 ± 7.6	0.4	50	0.66	(−3.5, 5.5)	<0.01
Logical memory I: immediate recall	12.1 ± 4.2	14.6 ± 3.8	−2.7	50	**0.01**	(−4.8, −0.7)	0.13
Logical memory II: delayed recall	9.9 ± 5.0	12.9 ± 4.3	−2.8	50	**0.006**	(−5.7, −1.0)	0.14
Satisfaction with life scale	13.5 ± 6.3	22.6 ± 7.3	−4.9	50	****<**10**^−^**^4^**	(−12.4, −5.2)	0.33
Verbal problem solving	11.6 ± 1.6	12.4 ± 1.6	−1.9	47	0.06	(−1.8, 0.1)	0.07
Visual selective learning task	113.0 ± 34.6	113.1 ± 35.4	−0.2	50	0.88	(−20.2, 17.3)	<0.01

*WASI, Wechsler Abbreviated Scale of Intelligence; FSIQ, full scale intelligent quotient; WTAR, Wechsler Test of Adult Reading. See Table 1 for other abbreviations*.

*^a^Mean and SD values were reported*.

*^b^*p*-values were obtained with age and years of education covariates. Bold face indicates *p* < 0.05*.

### Group Differences in Amygdala Connectivity

**Figure 1:**
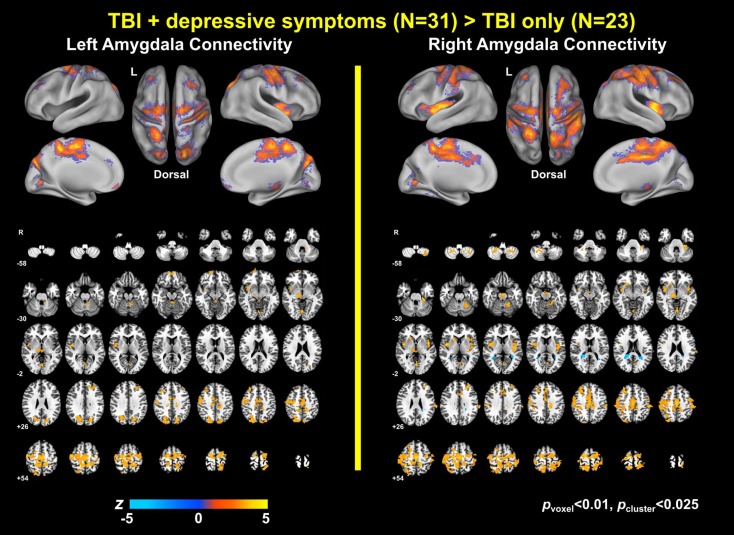
**Group comparison maps of amygdala connectivity**.

**Figure 2:**
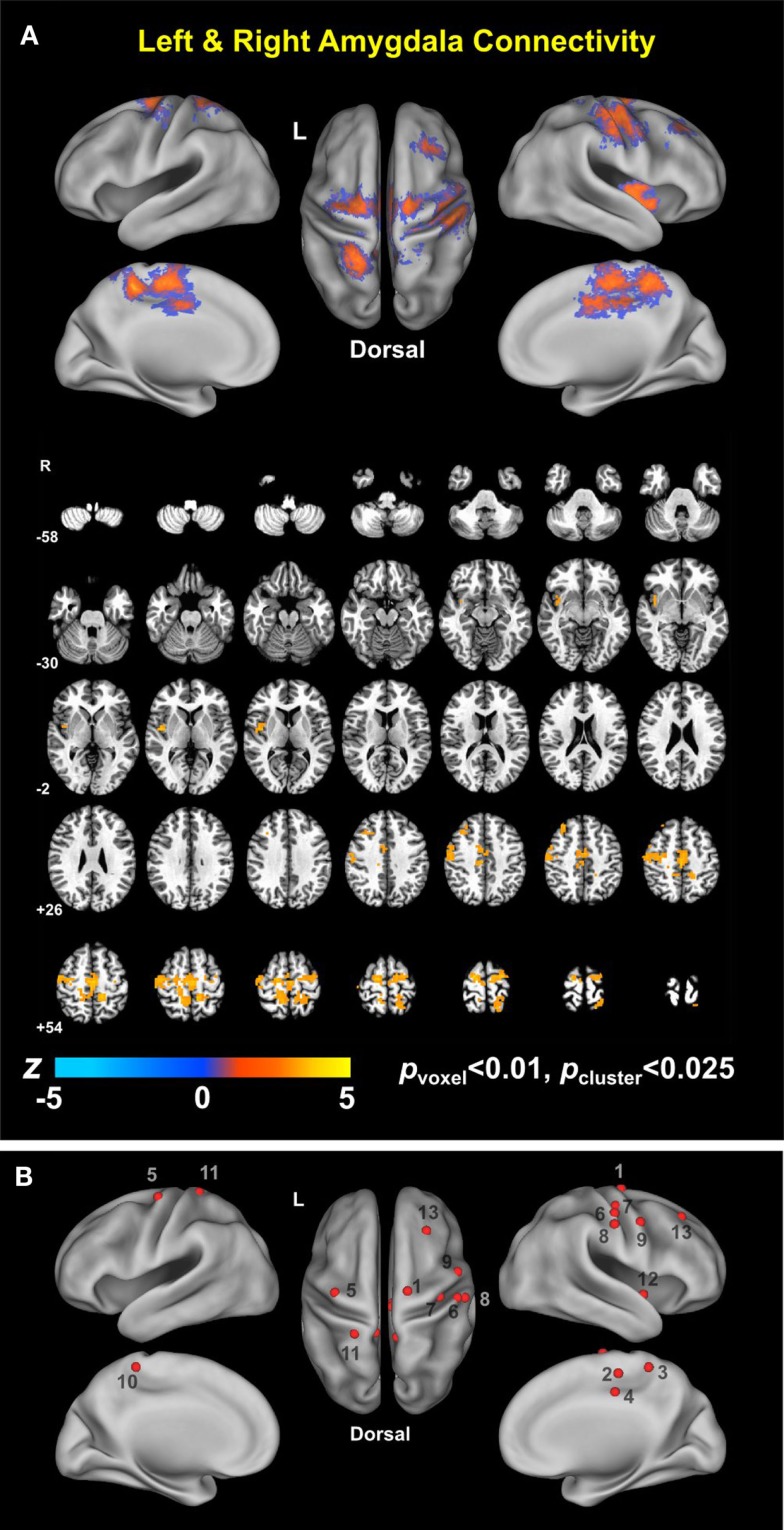
**Conjunction map of left and right amygdala connectivity (A) and foci of local peaks (B)**.

**Figure 3:**
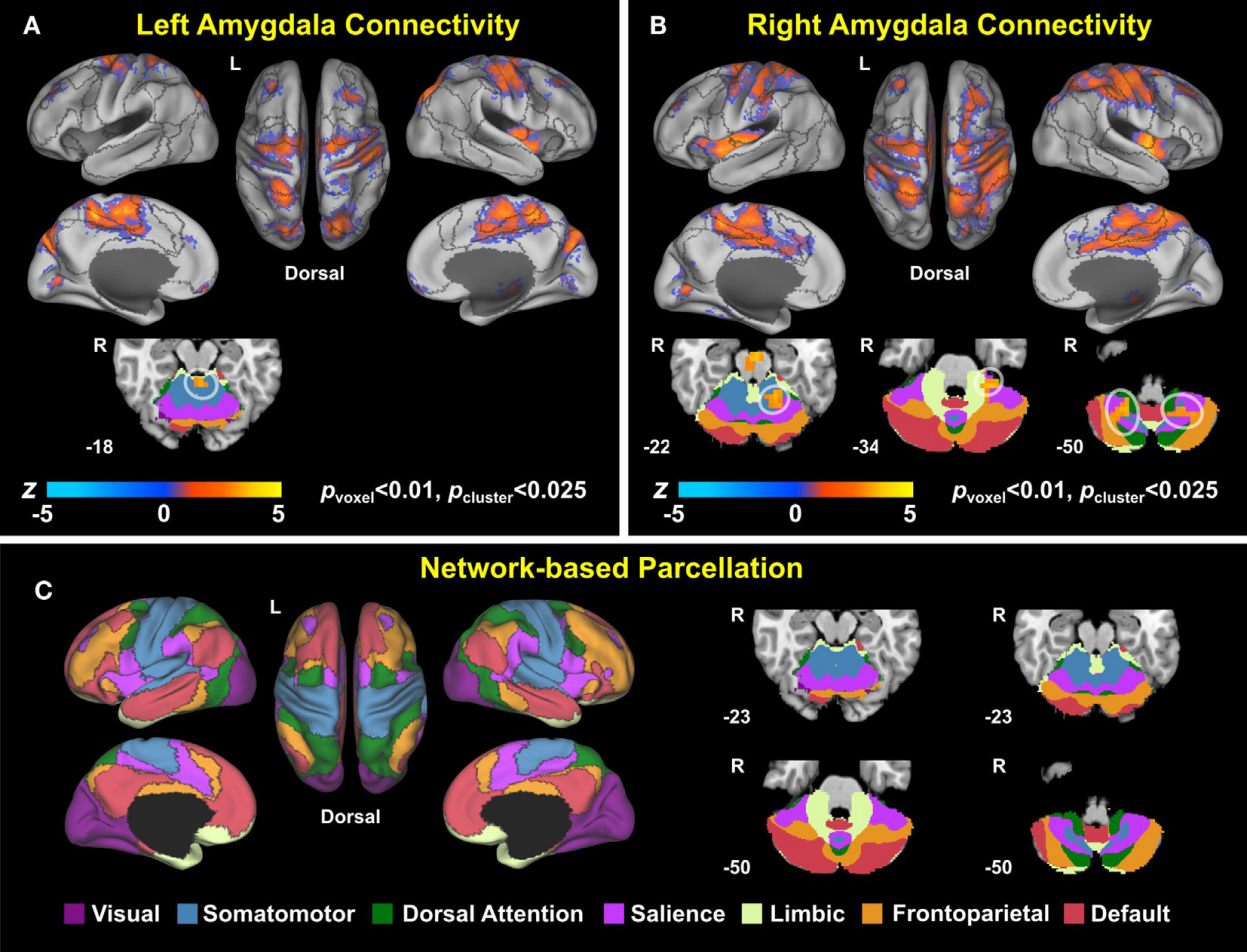
**Group comparison maps overlaid on network-based parcelation (106, 107)**. **(A)** Left amygdala connectivity, **(B)** right amygdala connectivity, **(C)** network-based parcellation. Note that we renamed the ventral attention network (VAN) in Buckner et al. (106) and Yeo et al. (107) as the salience network (SN) since (1) the VAN in Buckner et al. (106) and Yeo et al. (107) is an aggregate of multiple networks, including the SN and (2) most of the corresponding regions in the conjunction map fell onto the SN (35).

Group analysis revealed enhanced bilateral amygdala connectivity for the TBI-plus-depressive symptoms group relative to the TBI-only group across multiple regions except left amygdala connectivity with the left superior parietal lobule (SPL), right insula and right thalamus and right amygdala connectivity with the right thalamus at *p*_voxel_ < 0.01 and *p*_cluster_ < 0.025 (Figure 1). Spatial patterns of relatively enhanced amygdala connectivity of the TBI-plus-depressive symptoms group were fairly consistent over both amygdala connectivities though right amygdala connectivity showed stronger increases in the TBI-plus-depressive symptoms group over the TBI-only group. Conjunction analysis results (Figure 2) highlighted brain regions with consistently increased bilateral amygdala connectivity of the TBI-plus-depressive symptoms group over the TBI-only group. The regions showing increased connectivity for both left and right amygdala include the bilateral posterior midcingulate cortex, marginal sulcus, paracentral sulcus, precentral gyrus; the left SPL; the right dorsolateral prefrontal cortex (DLPFC), central sulcus, postcentral gyrus, and insula. Local peak coordinates of these regions and their *Z*-scores were listed in Table 4. Group comparison maps, including a network-based parcelation (Figure 3), revealed that alterations in amygdala connectivity of the TBI-plus-depressive symptoms group primarily occurred in the areas originally reported by Biswal et al. (118) and later labeled the somatomotor network (SMN) (119), the dorsal attention network (DAN) (112, 120), SN (35), and visual network (VN) (121). Relative increases in amygdala connectivity of the TBI group with depressive symptoms also occurred within areas of the limbic–cortical mood-regulating circuit (45), including the left DMPFC and right DLPFC and right thalamus (Figures 1–3).

**Table 4:** **Regions showing statistically significant group differences in both left and right amygdala connectivity (TBI-plus-depressive symptoms **>** TBI-only; *p*_voxel_ **<** 0.01; *p*_cluster_ **<** 0.025)**.

#	Region	Major cluster	*Z*^a^	x^b^	y^b^	z^b^
1	R Superior precentral sulcus	1 (298 voxels)	3.8	14	−14	66
2	Paracentral sulcus		3.7	2	−24	52
3	R Marginal sulcus		3.4	14	−46	58
4	Posterior midcingulate cortex		3.2	6	−22	42
5	L Precentral gyrus		3.1	−38	−10	62
6	R Postcentral gyrus	2 (106 voxels)	3.4	54	−14	54
7	R Central sulcus		3.3	38	−14	54
8	R Postcentral sulcus		3.2	54	−18	42
9	R Precental gyrus		3.1	50	2	46
10	L Marginal sulcus	3 (84 voxels)	3.7	−10	−42	58
11	L Superior parietal lobule		2.8	−22	−42	62
12	R Insula	4 (26 voxels)	2.8	40	−2	−2
13	R Dorsolateral prefrontal cortex	5 (24 voxels)	3.2	30	30	46

*L, Left; R, Right*.

*^a^Local maximum whose coordinate was closest to the center of mass*.

*^b^The Montreal Neurological Institute (MNI) space (89)*.

### Association of Amygdala Connectivity with the Buckley BDI Factors of the TBI-Plus-Depressive Symptoms Group

**Figure 4:**
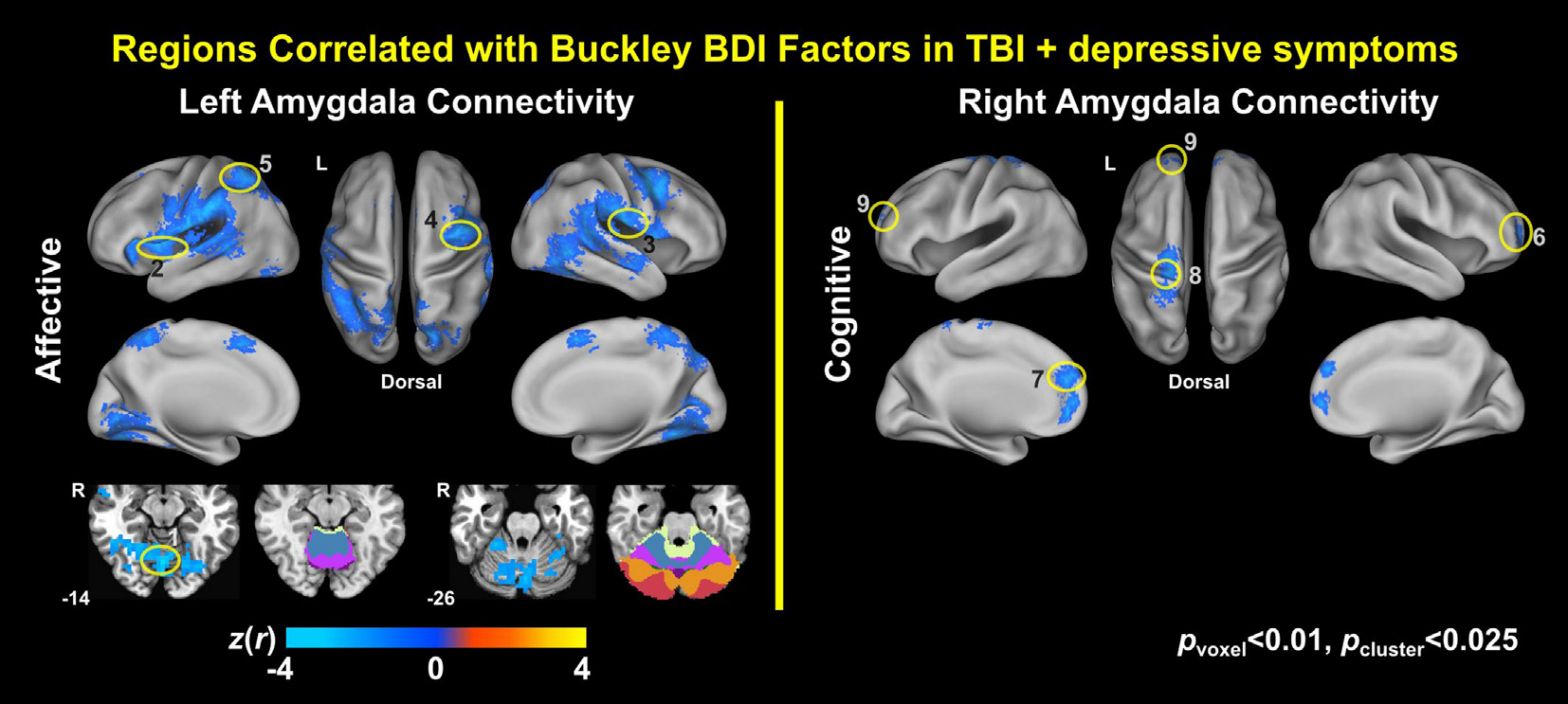
**Correlation maps of amygdala connectivity and the Buckley BDI factors within the TBI group with depressive symptoms**.

**Figure 5:**
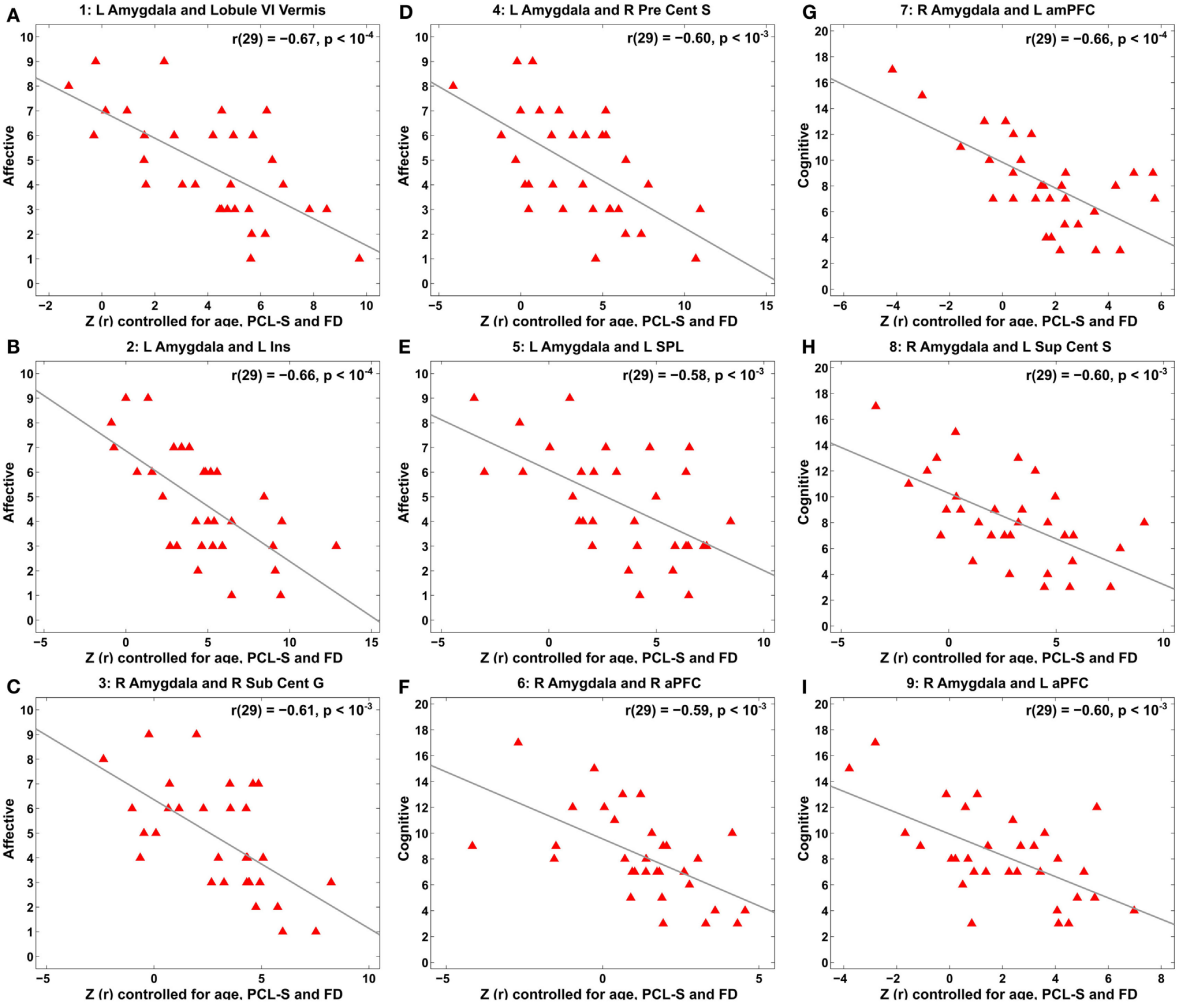
**Scatter plots for correlation between amygdala connectivity and the Buckley BDI factors within the TBI group with depressive symptoms at each of the selected nine local peaks in Figure 4**. **(A–E)** The Affective factor versus left amygdala connectivity in the cerebellar lobule VI vermis **(A)**, left insula **(B)**, right subcentral gyrus **(C)**, right precentral sulcus **(D)**, and left superior parietal lobule **(E)**, respectively. **(F–I)** The Cognitive factor versus right amygdala connectivity in the right anterior prefrontal cortex **(F)**, left anterior medial prefrontal cortex **(G)**, left superior central sulcus **(H)**, and left anterior prefrontal cortex **(I)**, respectively.

Voxel-wise correlation analysis of amygdala connectivity with each of the Buckley BDI factors (i.e., cognitive, affective, and somatic) exhibited dissociable spatial patterns over the whole brain within the TBI-plus-depressive symptoms group (Figure 4). Overall, only cognitive and affective factors were associated with amygdala connectivity among the TBI individuals with depressive symptoms (*p*_voxel_ < 0.01; *p*_cluster_ < 0.025), and such statistically significant correlations occurred in the regions that are part of the DMN, DAN, SN, SMN, FPCN, and VN. Specifically, the cognitive factor was negatively correlated with right amygdala connectivity in the bilateral aPFC and anterior medial prefrontal cortices (amPFC); left superior central sulcus. The affective factor was negatively correlated with left amygdala connectivity in the bilateral lingual gyri, subcentral cortices, superior temporal cortices, middle temporal complexes, marginal sulci and dorsal anterior cingulate cortices; left insula and SPL; right precentral sulcus and DLPFC. Negative association between the affective factor and left amygdala connectivity was also occurred in the cerebellar lobule VI Vermis and bilateral cerebellar lobules VI (hemisphere). Scatter plots for BDI factors versus amygdala connectivity controlled for age, PCL-S, and FD at selected nine local peak coordinates in Table 5 confirmed that statistically significant correlations did not erroneously occur by outliers (Figure 5).

**Table 5:** **Selected regions from the maps for correlations between amygdala connectivity and the Buckley BDI factors (*p*_voxel_ **<** 0.01; *p*_cluster_ **<** 0.025)**.

#	Seed	Factor	Region	*Z*^a^	x^b^	y^b^	z^b^
1	L amygdala	Affective	Lobule VI Vermis	4.3	2	−70	−14
2	L amygdala	Affective	L Insula	−4.2	−38	2	6
3	L amygdala	Affective	R Subcentral gyrus	−3.8	70	−10	14
4	L amygdala	Affective	R Precentral sulcus	−3.6	42	−2	46
5	L amygdala	Affective	L Superior parietal sulcus	−3.5	−34	−58	62
6	R amygdala	Cognitive	R Anterior prefrontal cortex	−3.6	22	50	6
7	R amygdala	Cognitive	L Anterior medial prefrontal cortex	−4.2	−10	50	22
8	R amygdala	Cognitive	L Superior central sulcus	−3.7	−14	−30	70
9	R amygdala	Cognitive	L Anterior prefrontal cortex	−3.7	−10	58	2

*L, Left; R, Right*.

*^a^Minimum *Z*-statistic values of the regions*.

*^b^The Montreal Neurological Institute (MNI) space (89)*.

### Connectivity Over 268 Putative Functional Nodes

**Figure 6:**
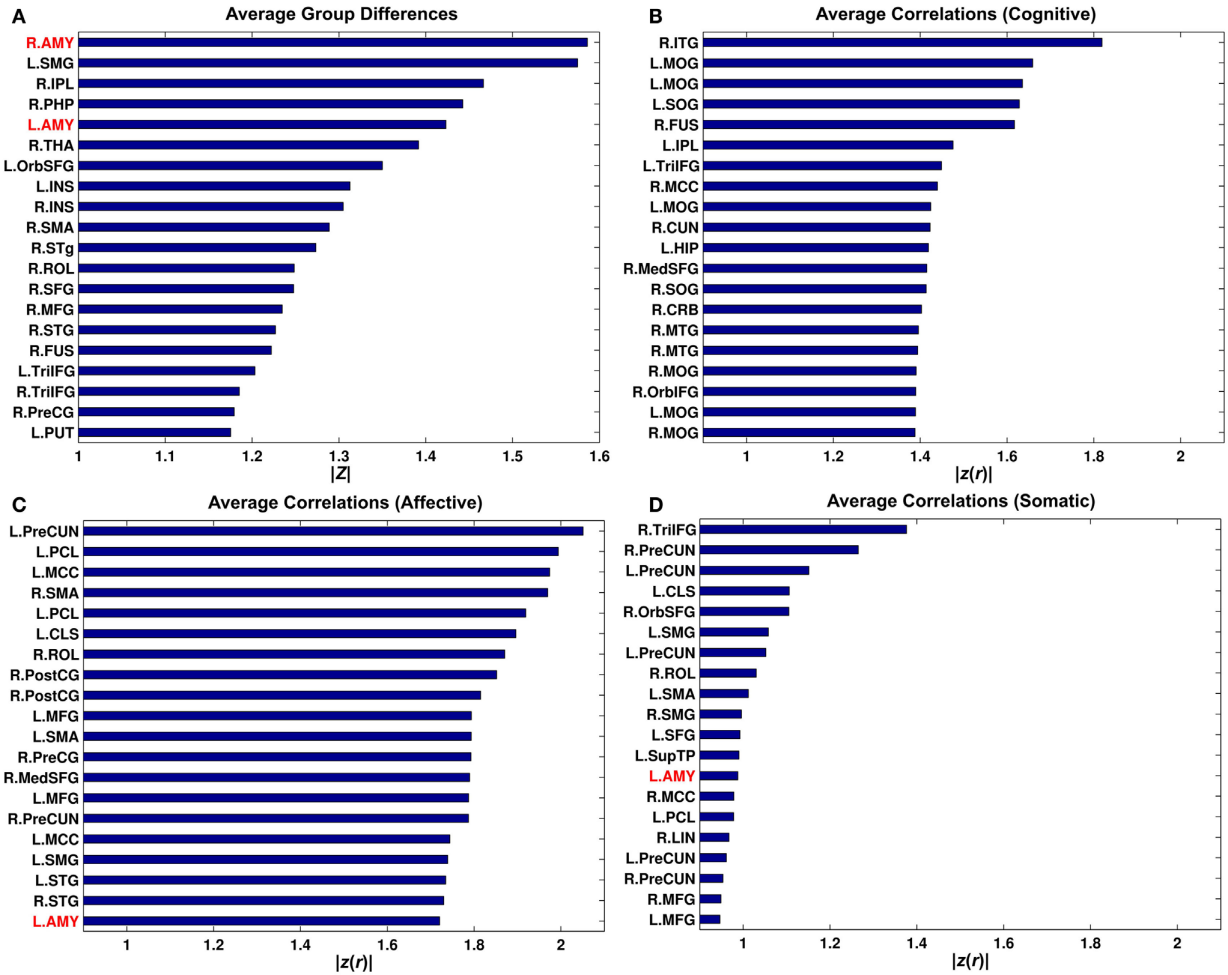
**Bar graphs for average group differences (A) and average correlation between connectivity strength and the Buckley BDI sub-scores (B-D) over 268 putative functional nodes**. Only top 20 nodes for each of the measures were shown. See Cao et al. (109) for abbreviations for node names.

Bar graphs for average group differences in connectivity and correlations between connectivity and BDI sub-factors over the rest of the putative functional nodes were obtained by seeding each of the 268 putative functional nodes. This analysis revealed that the amygdala seed connectivity showed pronounced average group differences relative to other seeds (Figure 6). However, the right amygdala seed was not an important driver of correlations with the BDI cognitive factor and correlations with the BDI affective factor can be better represented via other seeds (e.g., the left precuneus).

### Power Analysis Results

**Figure 7:**
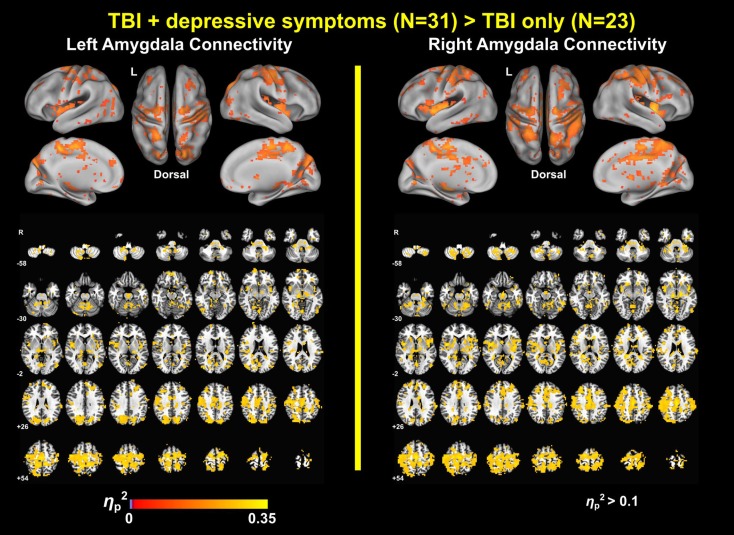
**Effect size maps for group differences in amygdala connectivity**.

**Figure 8:**
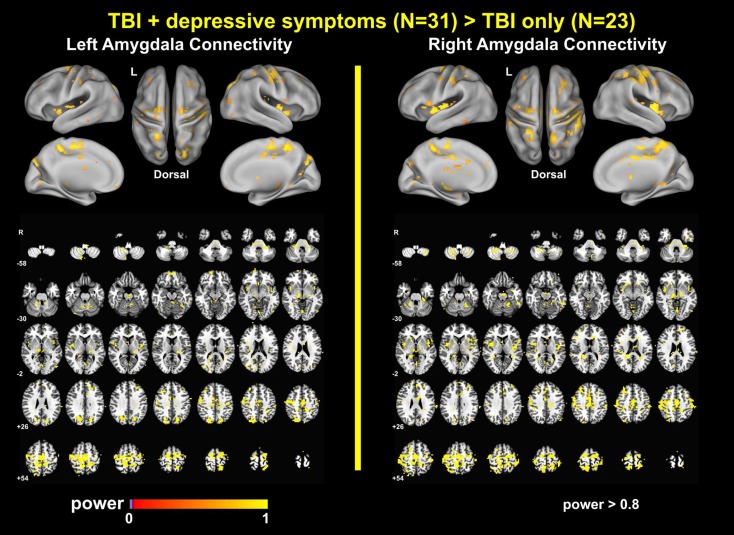
**Observed power for group differences in amygdala connectivity**.

The power analyses demonstrated that our findings have sufficiently reached both statistical significance and statistical power (Figures 7 and 8). The effect size maps for group differences in amygdala connectivity (Figure 7) revealed that the patterns of voxels whose group differences in amygdala connectivity accounted for more than 10% of total variance were similar to those of statistical significance at *p*_voxel_ < 0.01 and *p*_cluster_ < 0.025 (Figure 1). Again, the patterns of observed power values at >0.8 for group comparisons of left and right amygdala connectivity (Figure 8) were similar to those of statistical significance at *p*_voxel_ < 0.01 and *p*_cluster_ < 0.025 (Figure 1).

### Control Analysis Results

#### Somatomotor Connectivity

Somatomotor connectivity maps for the TBI-plus-depressive symptoms and TBI-only groups (Figure S1 in Supplementary Material) showed that both groups had strong somatomotor connectivity in the bilateral somatomotor cortices and supplementary motor cortex, which was similar to somatomotor connectivity of healthy individuals (118, 119).

#### Group Comparisons with Healthy Individuals

Relative to the healthy individuals, the age-matched TBI individuals had higher depressive symptom severity (Table S1 in Supplementary Material), lower satisfaction with life scale scores and poorer performance on multiple neuropsychological assessments (Table S2 in Supplementary Material). Group comparisons of PCC and aPFC connectivity measures with those of healthy individuals revealed that both the TBI-plus-depressive symptoms and TBI-only groups showed relative reductions in PCC and aPFC connectivity (Figure S2 in Supplementary Material). There were no statistically significant group differences in PCC or aPFC connectivity between the TBI sub-groups at *p*_voxel_ < 0.01 and *p*_cluster_ < 0.025. Group comparisons of amygdala connectivity in the age-matched TBI sub-groups with the healthy group demonstrated that both groups showed relative reductions in amygdala connectivity with more pronounced reductions in the TBI-only group (Figure S3 in Supplementary Material). Note that, in these group comparisons with the healthy group, there were no statistically significant effects of age on any of the assessed neuropsychological test scores at *p* < 0.05 or any of the obtained connectivity measures at *p*_voxel_ < 0.01 and *p*_cluster_ < 0.025.

#### Amygdala Connectivity in Civilians Versus Veterans Within the TBI-Plus-Depressive Symptoms Group

Civilian versus veteran group comparisons of amygdala connectivity were adjusted to control for age, PCL-S, and FD (Figure S4 in Supplementary Material) and did not show statistically significant differences between civilians and veterans within the TBI-plus-depressive symptoms group at *p* < 0.05 in each of the selected nine regions in Figure 4.

#### Effects of Comorbid PTSD Symptom Severity on Amygdala Connectivity

Statistically significant (*p*_voxel_ < 0.01 and *p*_cluster_ < 0.025) effects of PTSD-related covariates occurred by the PCL-S scores and age by PCL-S interaction in the TBI-plus-depressive symptom group for right amygdala connectivity (Figure S5 in Supplementary Material). However, the spatial extent of these PTSD-related effects was small compared to the group differences in amygdala connectivity demonstrated in Figures 1–3.

#### Effects of Estimated Injury Severity on Depressive Symptom Severity and Amygdala Connectivity

There were no systematic effects of estimated injury severity on the TBI participants’ BDI-II total scores and amygdala connectivity at selected four regions from Table 4 (Figure S6 in Supplementary Material). Group comparison maps for amygdala connectivity of the TBI participants with *probable* mild TBI only were essentially similar to results for the full TBI participants at *p*_voxel_ < 0.01 and *p*_cluster_ < 0.025 (Figure S7A in Supplementary Material). Again, group comparison maps for amygdala connectivity of the resampled TBI sub-groups, whose overall group differences magnitude in amygdala connectivity corresponded to the median value among those of the entire resampled pool, essentially replicated the patterns of amygdala connectivity differences for the full TBI participants at *p*_voxel_ < 0.01 and *p*_cluster_ < 0.025 (Figure S7B in Supplementary Material). The magnitude of overall group differences in amygdala connectivity of the *probable* mild TBI sub-groups did not fall outside the 2.5–97.5th percentile intervals of group differences obtained from 5,000 resampled groups, suggesting that there were no “significant” effects of estimated injury severity on the amount of overall group differences in amygdala connectivity.

## Discussion

In summary, we confirmed that depressive symptoms altered amygdala connectivity in the individuals with TBI over multiple brain regions and networks. Relative increases in amygdala connectivity within the TBI group with depressive symptoms primarily occurred in the regions that are part of the SN, SMN, DAN, and VN. Alterations in amygdala connectivity also occurred within the areas of the limbic–cortical mood-regulating circuit (45) that includes the DMPFC, DLPFC, and thalamus. Amygdala connectivity also revealed spatially dissociable patterns of correlation with symptom severity according to the Buckley subtypes (Cognitive and Affective) of depressive symptoms (67), further indicating the utility of network-based rsfMRI as a sensitive tool for assessing the relationships between brain networks and depressive symptoms in TBI individuals.

### Advantages of Large-Scale Network Approaches to Comorbid Depressive Symptoms in TBI

This is the first study that identified a connectivity-based biomarker for depressive symptoms among TBI individuals. We characterized the spatial patterns of altered amygdala connectivity among TBI individuals with depressive symptoms in the context of large-scale networks such as the DAN, DMN, SN, SMN, and VN. Large-scale network approaches to TBI studies have been increasingly favored because one of the primary injury mechanisms for TBI is DAI and DAI disrupts structural and functional connectivity [see Sharp et al. (17) for a review]. Recently, large-scale network approaches have also been appearing with greater frequency in the depression literature (28, 29, 31, 34, 37, 122, 123). The utility of large-scale network approaches for evaluating depression is promising because depression is well known for its heterogeneity of symptoms, systems, regions, and biochemical influences (30). Prior attempts to identify localized brain regions responsible for depressive symptoms have met with limited success. Based on previous demonstrations of the utility of applying large-scale network approaches in TBI and depression studies, our findings demonstrated potential advantages of large-scale network approaches to the evaluation of comorbid depressive symptoms among TBI individuals. As demonstrated in Figure 3, the patterns of widely spread alterations in amygdala connectivity of the TBI individuals with comorbid depressive symptoms were more precisely characterized when we compared the group comparison maps (Figures 1 and 2) with functional connectivity-based parcelation maps of the healthy brain (106, 107). Thus, we suggest that taking a large-scale network perspective, as opposed to region-by-region assessments, can augment the ability to understand altered patterns of connectivity in future studies of both civilians and veterans with TBI and comorbid depression. Furthermore, utilizing graph theory to assess the brain network topology of these TBI individuals with depressive symptoms may also be of great interest and may further enhance our understanding of network alterations among these participants, since our findings suggest that depressive symptoms may disrupt information processing between the amygdala and other brain networks.

### Findings in Relation to Previous Studies

#### Patterns of Altered Amygdala Connectivity

The amygdala is densely interconnected with the rest of the brain (49). Previous resting-state functional connectivity in healthy individuals has demonstrated rich connections from several brain areas with the amygdala (124). Specifically, the amygdala is functionally connected with the cingulate gyrus, precuneus, superior, middle and inferior frontal gyri, superior, middle and inferior temporal gyri, precentral gyrus, superior and inferior parietal lobules, angular gyrus, lateral occipital lobe, lingual gyrus, fusiform gyrus, insula, hippocampus, caudate, thalamus, brain stem, pons, and cerebellum during the rest-state in healthy individuals. Amygdala connectivity in depressed individuals is altered in a variety of brain regions (29, 31). Thus, it was not surprising that alterations in the amygdala connectivity among TBI individuals with depressive symptoms occurred over multiple brain regions affiliated with the SN, DAN, SMN, and VN as well as the limbic–cortical mood-regulating network. It is, however, a novel finding and the specific areas involved were not previously identified in this population.

##### Alterations with the Salience Network

The SN (35), comprising the dorsal anterior cingulate cortex, anterior insula, temporal pole, presupplementary motor area, amygdala, putaman, periaqueductal gray, substantia nigra, ventral tegmental area, dorsomedial thalamus, and hypothalamus, is involved in filtering information to support behavior choice. In the depression literature, the SN has also been described as the AN because of the large overlapping regions between these networks. The AN consists of the anterior cingulate cortex, amygdala, hypothalamus, entorhinal cortex, nucleus accumbens, and other limbic structures, and the AN involved in emotion regulation and processing emotional stimuli. (125). An extensive literature describes alterations in AN activity in depression [see Johansen-Berg et al. (126) and Price and Drevets (127) for a review], and some previous studies have directly examined the AN in depression at rest (37, 128). Among the altered regions in the SN, the insula and the dorsal anterior cingulate cortex are of particular interest. Task activation studies demonstrated that the insula is associated with emotional responses to interoceptive sensory stimuli (129) and the dorsal anterior cingulate is involved in cognition, emotion regulation, and attention (130).

##### Alterations with the Dorsal Attention Network

The DAN is comprised of the superior parietal lobule, the middle temporal complex, and the frontal eye field (120). The DAN is involved in top-down control of attention in that it is associated with the control of spatial attention through selecting sensory stimuli according to internal goals or expectations and applying these toward making appropriate motor responses (131). No prior studies had directly investigated the DAN in depression at rest. However, previous studies in healthy individuals reported increased connectivity within the DAN and between the DAN and VN when the participants engaged in the reappraisal task relative to maintaining emotional responses, suggesting that the DAN may be critical in volitional emotion regulation (132). Furthermore, recent functional neuroimaging research revealed that enhanced sensory responses to emotional stimuli can gain prioritized access to awareness after competing for attentional resources (133). In this “emotional attention” process, the amygdala plays a crucial role by providing both direct and indirect top-down signals on sensory pathways (133, 134). Taken together, the altered amygdala connectivity with the DAN suggests that the interaction of emotion and attention was altered among the TBI individuals with depressive symptoms (134).

##### Alterations with the Somatomotor Network

Altered amygdala connectivity also occurred in the primary motor cortex, primary somatosensory cortex, and supplementary motor area (SMA) within the SMN in Yeo et al. (107). Though there are no prior reports of depression studies that investigated the SMN connectivity with the amygdala, a relevant animal study demonstrated that direct electrical stimulation of the amygdala can interrupt ongoing motor behaviors (135). In humans, the amygdala is functionally connected with the SMN (124, 136). Utilizing an emotional version of the stop-signal fMRI task, Sagaspe et al. (137) revealed that the amygdala involves motor inhibition by emotional signals through interaction with the SMA. Thus, altered amygdala connectivity with the SMN among TBI individuals with depressive symptoms may be associated with disrupted motor inhibition in response to emotional stimuli. Future task connectivity studies are required to support this possibility. Among the altered regions in the SMN, the SMA is of particular interest. The SMA was strongly altered along the midline in our study (Figures 1–3). Within the complex network perspective, the SMA together with the PCC serve as functional core hubs that balance segregation and integration of local brain systems to maintain the small-world architecture (136). In this vein, it would be interesting to assess if altered amygdala connectivity with the SMA leads to imbalance between segregation and integration associated with among TBI individuals with depressive symptoms in future studies.

##### Alterations with the Visual Network

Within the VN, the parieto-occipital sulcus and lingual gyrus were altered (Figures 1–3). The amygdala is extensively connected with VN both structurally (138) and functionally (124). Previous depression studies also demonstrated abnormal connectivity in brain regions within the VN (46, 122, 139, 140). Based on an extensive supporting literature, Pessoa and Adolphs (141) proposed that affective visual signals flow not only through fast and automatic pathway from the visual cortex to the amygdala, but also through slow, diverse pathways from the pulvinar to the amygdala and back to the pulvinar via distributed cortical regions. Furthermore, previous connectivity studies in depression that reported abnormalities in the visual cortex have also revealed abnormalities in both the amygdala and other brain regions (46, 122, 140). Thus, altered amygdala connectivity within the VN in concert with other resting-state networks suggests that depressive symptoms in TBI may disrupt a modulatory role of the amygdala in evaluating affective visual stimuli such as salience, significance, ambiguity, and unpredictability (142–145) via a wide array of networks. Future studies utilizing task-state functional connectivity and behavioral assessment will be required to confirm this hypothesis.

##### Alterations with the Other Networks

Altered amygdala connectivity also occurred in the regions within the DMN (the left DMPFC), the FPCN (the right DLPFC), and the thalamus (Figures 1, 3, 7–8). The DMPFC, which is structurally connected with the amygdala (49), may mediate the alterations of amygdala connectivity with the DMN and FPCN in our study since Sheline et al. (37) demonstrated that connectivity increases in the DMN, FPCN, and AN overlap within the DMPFC. In the broader context of the depression literature, the amygdala, DLPFC, and thalamus serve as parts of the limbic–cortical mood-regulating network where the coordinated interactions within the limbic–cortical network are critical to the integration of mood regulation and mood-related motor, cognitive and somatic behaviors (45).

##### Alterations with the Cerebellum

Depressive symptoms in TBI also altered amygdala connectivity with the cerebellum (Figures 1–3). Traditionally, the cerebellum was thought to involve in motor coordination. However, converging, recent evidence has also suggested that the cerebellum may also be important in both emotion and cognition (146, 147). Furthermore, recent resting-state functional connectivity evaluated in healthy individuals has demonstrated interactions of the cerebellum with entire resting-state networks within the cerebral cortex (106). Indeed, previous resting-state fMRI studies in depression have revealed abnormalities in the cerebellum (46, 139, 140, 148–152). Alterations in amygdala connectivity with the cerebellum among individuals with depression also have been reported (46, 140). A novel finding on altered amygdala connectivity with the cerebellum in our study is that cerebellar regions that showed altered amygdala connectivity fell onto the DAN, SMN, and SN which showed alterations in the cerebral cortex. This consistency between the cerebellum and cerebral cortex in altered networks with amygdala connectivity in TBI with depressive symptoms reinforces the idea that depressive symptoms in TBI disrupt coordinating roles of the amygdala with multiple resting-state networks over the whole brain.

##### Elevated Amygdala Connectivity in TBI Individuals with Depressive Symptoms

Relative increases in amygdala connectivity among TBI individuals with depressive symptoms indicate that depressive symptoms in TBI may increase neural resource recruitment relevant to information processing between the amygdala and other brain regions in emotion responses. Future task-state functional connectivity and metabolism studies may confirm this hypothesis. Increases in amygdala connectivity in TBI with depressive symptoms appear to be inconsistent with findings from previous resting-state amygdala connectivity studies in major depressive disorder (40, 43, 46, 48, 122). Such discrepancies might be attributed to potential effects of interactions between other pathology (TBI in this study) and depressive symptoms on resting-state functional connectivity. For example, depressed individuals with Parkinson’s disease showed increased amygdala connectivity over non-depressed individuals with Parkinson’s disease whereas depressed individuals with Parkinson’s disease showed both increased and decreased amygdala connectivity over healthy individuals (153). Furthermore, Maller and colleagues (54, 56) demonstrated that cortical volumes and DTI measures were different among individuals with TBI-depression, TBI-no-depression, and no-TBI-depression, suggesting the effects of interactions between depression and TBI on the brain.

#### Correlations of Amygdala Connectivity with Subtypes of Depressive Symptoms

Correlation analysis of amygdala connectivity and the Buckley BDI-II factors exhibited spatial patterns of amygdala connectivity specific to cognitive and affective factors of depressive symptoms (Figures 4 and 5). Our correlation analysis results support potential utility of amygdala connectivity for TBI individuals with depressive symptoms in a clinical standpoint. Depression is a complex psychiatric disorder, and individuals with depression show heterogeneous symptoms ranging from somatic symptoms, panic attacks, obsessive behavior, ruminations, poor concentration to suicidal thoughts (154). Thus, stratifying individuals with heterogeneous depressive symptoms in an objective fashion based on amygdala connectivity may be useful for clinicians to plan more effective and individualized treatments for these heterogeneous patients. In this vein, our findings in Figures 4 and 5 further extended previous studies on neuroimaging correlates of total depressive symptom severity in TBI (40, 41, 52, 54, 56, 59).

We were also able to identify sensitive neuroimaging biomarker for subtypes of depressive symptoms with greater regional specificity than previous DTI study (58). In contrast to Strain et al. (58) that showed spatially overlapped correlation patterns across subtypes of depressive symptoms in concussion, our study demonstrated spatially dissociable patterns of amygdala connectivity according to subtypes of depressive symptoms. Specifically, the cognitive factor was correlated with right amygdala connectivity in the aPFC, amPFC, and left superior central sulcus of the DMN and SMN (Figure 4). The affective factor was correlated with left amygdala connectivity in the cerebral areas of the SN, DAN, VN, SMN, and FPCN and the cerebellum area of the SMN, FPCN, and SN (Figure 4).

One could wonder why statistically significant correlations between the Buckley cognitive factor and amygdala connectivity occurred in the areas of the SMN. Note that the Buckley BDI-II cognitive factor includes test items for sadness, pessimism, past failure, guilty feelings, punishment feelings, self-dislike, self-criticalness, suicidal thoughts, and worthless (Table 2). Thus, we should not confuse the Buckley cognitive factor with executive function, working memory, or reasoning in the context of cognitive neuroscience.

Amygdala connectivity was associated with only the Buckley cognitive and affective factors. This finding may be explained by differences in sensitivity to depressive severity over the Buckley factors. A previous study that investigated optimal BDI items for discriminating depressive severity in 335 medical patients, 16% of whom were neurologically impaired, identified that the items related to past failure, self-dissatisfaction, punishment feelings, suicidal thoughts, crying, loss of social interest, and indecisiveness well discriminated depressive severity in the medical patients (155). Three of these seven items are related to the Buckley BDI-II cognitive factor and the other four items are related to the Buckley BDI-II affective factor. Thus, cognitive and affective factors might have sufficient sensitivity to depressive symptom severity that enables to yield statistically significant correlations with amygdala connectivity across our TBI participants with depressive symptoms.

Although our findings of relationships between amygdala connectivity and the Buckley BDI sub-scores were interesting, the data-driven connectivity analysis over 268 nodes revealed that these relationships could be better represented via other seeds (Figure 6; Figure S8 in Supplementary Material). Furthermore, we observed positive associations between the Buckley BDI cognitive factor and connectivity with most of the other seeds than the amygdala (Figure S8 in Supplementary Material). These results demonstrates limitations of seed-based approaches that warrant complex network approaches in future directions to more comprehensively characterize the relationships between network topology and the BDI sub-scores in chronic TBI.

### Limitations and Future Research

The present study has several limitations. First, we assessed depressive symptom severity based on self-reports from the TBI participants. Although the BDI-II is one of the most widely used measures for depressive symptoms and shows good reliability (62), frequent impairments in self-awareness among TBI individuals could bias the reported depressive symptoms obtained in the BDI-II questionnaire (156). Thus, our findings should be interpreted with caution due to this limitation and clinical diagnosis for depression should not be made based exclusively on participants’ BDI-II total scores. Second, similar to the first limitation, we retrospectively identified the existence of brain injury, and estimated initial injury severity and the duration of LOC from the OSU TBI screening form (71), as opposed to reporting the gold standard measure for initial injury severity (i.e., GCS) and other clinical information from the acute-care, inpatient facilities where they were hospitalized sometimes multiple years ago. Thus, our study participants may be best characterized as individuals with a self-identified, probable history of TBI. Although we made our best efforts to identify TBI and estimate the clinical information at the acute stage such as initial injury severity and the duration of LOC utilizing the OSU TBI-ID method, whose validity and reliability have been demonstrated (71, 72), our findings should be interpreted within this limitation. Third, our TBI groups are a mixture of individuals with *probable* mild, *probable* moderate, and *probable* severe TBI, which may not be ideal, particularly for studies in sub-acute (3–6 months post-injury) and short-term chronic (6 months–2 years post-injury) stages of TBI. However, at the long-term chronic stage of TBI (>2 years post-injury), initial injury severity often plays less critical role in characterizing TBI individuals at the time of study (157, 158) and chronic TBI studies occasionally have reported with a mixture of different injury severity (10, 12, 13, 15, 19, 55, 158). In our case, there were no systematic effects of estimated injury severity on the BDI-II total scores or amygdala connectivity (Figures S6 and S7 in Supplementary Material). Nonetheless, care should be taken in the interpretations of our findings as injury severity was retrospectively estimated. Fourth, we discussed our findings in the context of previous resting-state functional connectivity studies in depressive individuals without other comorbid neurological conditions because no previous resting-state functional connectivity studies in TBI with comorbid depressive symptoms were reported. We do not know whether the patterns of altered amygdala in TBI individuals with comorbid depressive symptoms are similar to those of depressed individuals without TBI. This is a complex issue, due to the differences that likely exist between depressive symptoms in uninjured individuals and those who suffer from TBI-related symptoms. It may be possible that future studies that include additional groups of healthy individuals and individuals with depressive symptoms but without TBI would be required to address this concern. Fifth, one could argue that our findings may be driven by the effects of PTSD symptom severity on amygdala connectivity because of the frequently linked comorbidity of depression and PTSD in TBI (104) and statistically significant group differences in PTSD symptom severity in our groups. However, we included PTSD symptom severity and its interactions with other factors as covariates. Furthermore, (1) spatial extent of the regions that showed statistically significant effects of these covariates and (2) overlaps between these regions and the regions that showed group differences in amygdala connectivity were minimal (Figure S5 in Supplementary Material). Thus, our findings on altered amygdala connectivity in the TBI-plus-depressive symptoms group were primarily associated with depressive symptom severity rather than PTSD symptom severity. Sixth, we were not able to measure other potential confounding factors such as genetic predisposition, environment, other anxiety disorders, and chronic pain levels present in the participants. Especially in future amygdala connectivity studies in TBI with depressive symptoms, gene, and environment should be measured because of reported influences of genes and environment on amygdala connectivity in depression (159). Seventh, we investigated amygdala connectivity with the amygdala as a single entity. In fact, the amygdala consists of structurally and functionally distinct nuclei. For example, laterobasal, centromedial, and superficial amygdala subdivisions have different functional connectivity patterns in healthy individuals (124). Future amygdala connectivity studies with amygdala subdivisions may be further elucidate alteration patterns in TBI with depressive symptoms. Lastly, our sample consisted of individuals who were often multiple years post-TBI, and individuals who had experienced multiple TBI incidents. This leads to the possibility that connectivity alterations may be different in individuals who are evaluated closer in time to their TBI incident.

Our future studies include an assessment of if and how altered amygdala connectivity in chronic TBI with comorbid depressive symptoms may be reorganized following rehabilitation. We will also further identify alteration patterns utilizing graph theory. Finally, we will address concerns discussed above in our future communications.

## Conclusion

In conclusion, we demonstrated pronounced, widespread alterations in amygdala connectivity among chronic TBI individuals with comorbid depressive symptoms. Such widespread alterations in amygdala connectivity indicate alterations in modularity roles of the amygdala in emotion processes among TBI individuals with depressive symptoms. Amygdala connectivity also showed spatially dissociable patterns of correlation with symptoms severity according to different subtypes of depressive symptoms. Taken together, our findings suggest that amygdala connectivity may be a potentially effective neuroimaging biomarker for comorbid depressive symptoms among individuals with chronic TBI.

## Author Contributions

All authors were involved in the conceptualization and design of this study, the drafting and revising of the manuscript, and the interpretation of the data. KH substantially contributed to the data analysis.

## Disclaimer

The views and opinions expressed in this article are those of the authors and do not reflect the official policy or position of the Department of the Army, Department of the Air Force, Department of Defense, or United States Government.

## Conflict of Interest Statement

The authors declare that the research was conducted in the absence of any commercial or financial relationships that could be construed as a potential conflict of interest.

## Footnotes

^1^http://afni.nimh.nih.gov/pub/dist/doc/program_help/afni_proc.py.html

^2^http://www2.warwick.ac.uk/fac/sci/statistics/staff/academic-research/nichols/scripts/fsl/DVARS.sh

^3^http://www.brainmap.org/icbm2tal/
