# Supplementary material for: Altered Amygdala Connectivity in Individuals with Chronic Traumatic Brain Injury and Comorbid Depressive Symptoms
Source: Front Neurol. 2015 Nov 4;6:231. doi: 10.3389/fneur.2015.00231 (PMC4631949; doi:10.3389/fneur.2015.00231)
Supplement: Supplementary file 1 [file table_1.doc]

**Table S1.** Demographics of the healthy individuals and the age-matched TBI sub-groups.

| Demographics | TBI-plus-depressive symptoms^a^ | TBI-only^b^ | Healthy | Stat | DF | *p*-values^c^ | CI | ES |
| --- | --- | --- | --- | --- | --- | --- | --- | --- |
| Number of participants | 21 | 16 | 17 | - | - | - | - | - |
| Age (years)^d^ | 32.5 ± 7.0 | 33.2 ± 8.8 | 27.7 ± 8.5 | 1.9, 1.8 | 30.8, 30.7 | 0.07, 0.08 | [-0.4, 10.1], [-0.6, 11.7] | 0.62, 0.62 |
| Eduction (years)^d^ | 15.4 ± 2.1 | 15.8 ± 1.9 | 14.9 ± 2.3 | 0.6, 1.2 | 32.4, 30.3 | 0.53, 0.24 | [-1.0, 1.9], [-0.6, 2.3] | 0.21, 0.40 |
| Gender (males, females) | 14, 7 | 11, 5 | 11, 6 | 0.8, 0.7 | - | 1.00, 1.00 | [0.3, 2.3], [0.2, 2.3] | 0.79 0.71 |
| BDI-II total^d^ | 21.2 ± 5.8 | 7.4 ± 3.9 | 3.6 ± 4.6 | 10.4, 2.6 | 36.0, 30.7 | <**10^-11^**, **0.01** | [14.2, 21.1], [0.8, 6.9] | 3.25, 0.87 |
| BDI-II Buckley cognitive^d^ | 8.0 ± 3.9 | 1.3 ± 1.3 | 0.9 ± 1.2 | 8.0, 0.8 | 24.8, 30.2 | **<10^-7^**, 0.41 | [5.3, 9.0], [-0.5, 1.3] | 2.33, 0.28 |
| BDI-II Buckley affective^d^ | 4.7 ± 2.0 | 1.9 ± 1.8 | 0.5 ± 1.2 | 8.2, 2.5 | 33.6, 25.5 | **<10^-8^**, **0.02** | [3.1, 5.2], [0.2, 2.5] | 2.48, 0.86 |
| BDI-II Buckley somatic^d^ | 8.5 ± 3.2 | 4.1 ± 2.2 | 2.2 ± 2.9 | 6.4, 2.1 | 35.3, 29.7 | **<10^-6^**, 0.05 | [4.3, 8.4], [<0.1, 3.7] | 2.02, 0.70 |
| Motion censored volumes (%)^d^ | 16.5 ± 12.9 | 13.4 ± 9.4 | 12.7 ± 13.4 | 457, 291.5 | - | 0.17, 0.49 | [-3.0, 10.4], [-4.2, 8.2] | -0.20, 0.11 |
| FD after censoring and trimming (mm)^d^ | 0.17 ± 0.05 | 0.15 ± 0.04 | 0.15 ± 0.04 | 0.8, -0.4 | 35.0, 31.0 | 0.43, 0.67 | [-0.02, 0.04], [-0.04, 0.02] | 0.25, -0.14 |

*Note*: See Table 1 for abbreviations and footnotes.
