# Supplementary material for: Altered Amygdala Connectivity in Individuals with Chronic Traumatic Brain Injury and Comorbid Depressive Symptoms
Source: Front Neurol. 2015 Nov 4;6:231. doi: 10.3389/fneur.2015.00231 (PMC4631949; doi:10.3389/fneur.2015.00231)
Supplement: Supplementary file 2 [file presentation_1.pdf]

Supplementary Figure Legends

Figure S1. Group statistic maps for somatomotor connectivity of the TBI sub-groups. Seeds were placed at the motor cortex (L: -41, -18, 59; R: 46, -19, 54) with a 5 mm radius sphere, respectively. Then the same general linear model for amygdala connectivity was used to obtain the group statistic maps from Fisher's Z-transformed correlation coefficients.

Figure S2. Group comparison maps for posterior cingulate cortex (PCC) and anterior prefrontal cortex (aPFC) connectivity of the age-matched TBI sub-groups relative to the healthy individuals. Seeds were placed at PCC (L: -7, -55, 27; R: 8, -48, 31) and aPFC (L: -36, 57, 9; R: 34, 52, 10) with a 5 mm radius sphere, respectively. Then the general linear model using within-group centered age covariates was used to obtain the group contrast maps from Fisher's Z-transformed correlation coefficients.

Figure S3. Group comparison maps for left and right amygdala connectivity of the age-matched TBI-plus-depressive symptoms group (A) and TBI-only group (B) relative to the healthy individuals.

Figure S4. Scatter plots for amygdala connectivity of civilians and veterans within the TBI group with depressive symptoms at each of the selected nine local peaks in Fig. 4. The I bars indicate the means and standard deviation of the civilian subgroup, the dotted horizontal bar is two standard deviations from the mean of the civilian subgroup and the solid horizontal bar in the veteran subgroup is the mean of the veteran subgroup. Filled triangles represents veterans whose amygdala connectivity strength was located outside the dotted horizontal bars. The *p*-values were obtained from the t-test.

Figure S5. Color maps for the effects of PTSD-related covariates on amygdala connectivity. PCL_D represents PCL-S scores for the TBI group with depressive symptoms.

Figure S6. Scatter plots for the BDI-II total scores (A) and amygdala connectivity of the TBI sub-groups (B-I) according to *estimated* injury severity. See Table 4 for the voxel coordinates of the selected four regions. See Fig. S4 legends for the details of the scatter plots (B-I).

Figure S7. Group comparison maps for amygdala connectivity of the TBI subgroups comprising of individuals with *probable* mild TBI only (A) and one instance of resampled groups by removing the same of number of *probable* mild TBI participants from the original TBI sub-groups (B). The average absolute value of Z-statistics for the group comparisons over the whole brain of the selected, resampled groups corresponds the median among those of the entire 5,000 resampled pool.

Figure S8. *Z*-statistic maps for group comparisons of connectivity strengths (A) and correlations between connectivity strengths and the Buckley BDI sub-scores (B-D) at each pair of the 268 putative functional nodes. Connectivity strengths are represented by Fisher's Z-transformed correlation coefficients followed by *Z*-score normalization.
