# Supplementary material for: Altered Amygdala Connectivity in Individuals with Chronic Traumatic Brain Injury and Comorbid Depressive Symptoms
Source: Front Neurol. 2015 Nov 4;6:231. doi: 10.3389/fneur.2015.00231 (PMC4631949; doi:10.3389/fneur.2015.00231)
Supplement: Supplementary file 3 [file table_2.doc]

**Table S2.** Neuropsychological assessment of the age-matched TBI sub-groups relative to the healthy individuals.

| Neuropsychological measure^a^ | TBI-plus-depressive symptoms | TBI-only | Healthy | *T* | DF | *p*-values^b^ | CI | η_p_^2^ |
| --- | --- | --- | --- | --- | --- | --- | --- | --- |
| Number of participants | 21 | 16 | 17 | *-* | - | - | - | - |
| Similarities | 37.2 ± 4.1 | 38.0 ± 4.1 | 38.1 ± 5.7 | -0.6, -0.1 | 51 | 0.57, 0.94 | [-3.9, 2.2], [-3.4, 3.1] | 0.01, <0.01 |
| Matrix reasoning | 28.1 ± 4.6 | 29.3 ± 4.2 | 30.2 ± 2.9 | -1.6, -0.7 | 51 | 0.12, 0.49 | [-4.7, 0.6], [-3.8, 1.8] | 0.05, 0.01 |
| WASI FSIQ-2 (current IQ) | 109.1 ± 10.6 | 112.9 ± 8.6 | 111.6 ± 14.7 | -0.7, 0.3 | 51 | 0.51, 0.75 | [-10.1, 5.1], [-6.8, 9.4] | 0.01, <0.01 |
| Digit span forward | 10.5 ± 2.3 | 10.8 ± 2.2 | 11.0 ± 2.6 | -0.7, -0.3 | 51 | 0.51, 0.77 | [-2.1, 1.0], [-1.9, 1.4] | 0.01, <0.01 |
| Digit span backward | 7.0 ± 2.2 | 7.6 ± 2.1 | 7.9 ± 2.2 | -1.3, -0.5 | 51 | 0.21, 0.62 | [-2.3, 0.5], [-1.9, 1.1] | 0.03, <0.01 |
| Color-word: Color naming (s) | 31.9 ± 8.1 | 28.9 ± 4.8 | 27.2 ± 5.7 | 2.2, 0.7 | 51 | **0.03**, 0.48 | [0.4, 9.0], [-2.9, 6.2] | 0.09, 0.01 |
| Color-word: Word reading (s) | 25.0 ± 7.1 | 22.1 ± 4.6 | 20.6 ± 4.3 | 2.4, 0.8 | 51 | **0.02**, 0.46 | [0.7, 8.1], [-2.5, 5.4] | 0.10, 0.01 |
| Color-word: Inhibition (s) | 59.6 ± 16.5 | 51.9 ± 12.0 | 49.6 ± 14.8 | 2.1, 0.5 | 51 | **0.04**, 0.65 | [0.4, 19.7], [-8.0, 12.7] | 0.08, <0.01 |
| Color-word: Inhibition/switching (s) | 67.5 ± 17.6 | 58.4 ± 13.3 | 57.0 ± 13.9 | 2.1, 0.3 | 51 | **0.04**, 0.79 | [0.5, 20.5], [-9.3, 12.1] | 0.08, <0.01 |
| Verbal fluency: Letter fluency, total correct | 39.0 ± 10.2 | 45.2 ± 10.0 | 42.2 ± 13.3 | -0.9, 0.8 | 51 | 0.38, 0.44 | [-10.6, 4.1], [-4.8, 10.9] | 0.02, 0.01 |
| Verbal fluency: Category fluency, total correct | 39.0 ± 8.7 | 47.8 ± 8.9 | 42.7 ± 9.1 | -1.3, 1.6 | 51 | 0.21, 0.11 | [-9.6, 2.1], [-1.2, 11.3] | 0.03, 0.05 |
| Verbal fluency: Category switching, total correct | 15.0 ± 2.8 | 14.9 ± 2.8 | 14.5 ± 2.3 | 0.6, 0.5 | 51 | 0.51, 0.62 | [-1.2, 2.3], [-1.4, 2.3] | 0.01, <0.01 |
| Verbal fluency: Category switching, total switching accuracy | 14.0 ± 2.9 | 13.9 ± 2.8 | 13.0 ± 2.5 | 1.2, 1.0 | 51 | 0.25, 0.34 | [-0.8, 2.9], [-1.0, 2.9] | 0.03, 0.02 |
| Sorting: Free sorting, confirmed correct sorts | 9.5 ± 2.5 | 10.2 ± 1.8 | 10.7 ± 2.1 | -1.7, -0.7 | 51 | 0.09, 0.50 | [-2.7, 0.2], [-2.0, 1.0] | 0.06, 0.01 |
| Sorting: Free sorting, description score | 36.0 ± 11.1 | 39.4 ± 7.6 | 42.5 ± 8.3 | -2.2, -1.0 | 51 | 0.04, 0.35 | [-12.7, -0.5], [-9.6, 3.4] | 0.08, 0.02 |
| Sorting: Sort recognition, description score | 36.9 ± 12.3 | 34.6± 11.2 | 42.4 ± 9.3 | -1.5, -2.0 | 51 | 0.14, **0.05** | [-12.7, 1.7], [-15.6, <-0.1] | 0.04, 0.07 |
| Sorting: Combined description score | 72.8 ± 21.9 | 74.0 ± 17.1 | 84.9 ± 15.5 | -2.0, -1.7 | 51 | 0.05, 0.10 | [-24.3, 0.1], [-23.9, 2.2] | 0.07, 0.05 |
| Trail making: Visual scanning (s) | 19.4 ± 5.0 | 16.6 ± 4.0 | 16.8 ± 4.1 | 1.8, -0.1 | 50 | 0.08, 0.94 | [-0.3, 5.6], [-3.3, 3.0] | 0.06, <0.01 |
| Trail making: Number sequencing (s) | 28.6 ± 8.3 | 25.8 ± 6.5 | 24.0 ± 7.9 | 1.8, 0.7 | 51 | 0.07, 0.52 | [-0.4, 9.6], [-3.6, 7.1] | 0.06, 0.01 |
| Trail making: Letter switching (s) | 28.0 ± 8.2 | 23.9 ± 4.5 | 24.9 ± 6.7 | 1.4, -0.4 | 51 | 0.16, 0.67 | [-1.3, 7.6], [-5.8, 3.7] | 0.04, <0.01 |
| Trail making: Number-letter switching (s) | 73.8 ± 28.4 | 63.8 ± 17.0 | 58.9 ± 15.0 | 2.1, 0.7 | 51 | **0.04**, 0.52 | [0.6, 29.1], [-10.3, 20.1] | 0.08, 0.01 |
| Trail making: Motor speed (s) | 22.9 ± 7.9 | 18.8 ± 5.3 | 19.3 ± 5.7 | 1.7, -0.2 | 51 | 0.10, 0.81 | [-0.7, 7.9], [-5.1, 4.1] | 0.05, <0.01 |
| Logical memory I: Immediate recall | 12.2 ± 4.4 | 15.4 ± 3.0 | 13.1 ± 5.0 | -0.7, 1.5 | 51 | 0.51, 0.14 | [-3.7, 1.9], [-0.7, 5.2] | 0.01, 0.04 |
| Logical memory II: Delayed recall | 9.4 ± 5.1 | 14.1 ± 3.2 | 12.5 ± 4.6 | -2.1, 1.0 | 51 | 0.04, 0.31 | [-6.0, -0.1], [-1.5, 4.7] | 0.08, 0.02 |
| Satisfaction with life scale | 15.0 ± 6.0 | 22.8 ± 6.9 | 27.2 ± 4.2 | -6.5, -2.2 | 51 | **<10^-7^**, **0.03** | [-16.0, -8.4], [-8.5, -0.4] | 0.45, 0.09 |
| Verbal problem solving | 11.7 ± 1.6 | 12.5 ± 1.7 | 12.7 ± 2.3 | -1.5, -0.3 | 51 | 0.13, 0.75 | [-2.3, 0.3], [-1.5, 1.1] | 0.05, <0.01 |
| Visual selective learning task | 114.0 ± 33.2 | 114.6 ± 32.4 | 128.5 ± 42.1 | -1.2, -1.1 | 49 | 0.23, 0.26 | [-38.8, 9.7], [-41.0, 11.3] | 0.03, 0.03 |

*Note*: See Tables 1, 3 for abbreviations and footnotes.
