# Supplementary figures and images for: Altered Amygdala Connectivity in Individuals with Chronic Traumatic Brain Injury and Comorbid Depressive Symptoms
Source: Front Neurol. 2015 Nov 4;6:231. doi: 10.3389/fneur.2015.00231 (PMC4631949; doi:10.3389/fneur.2015.00231)

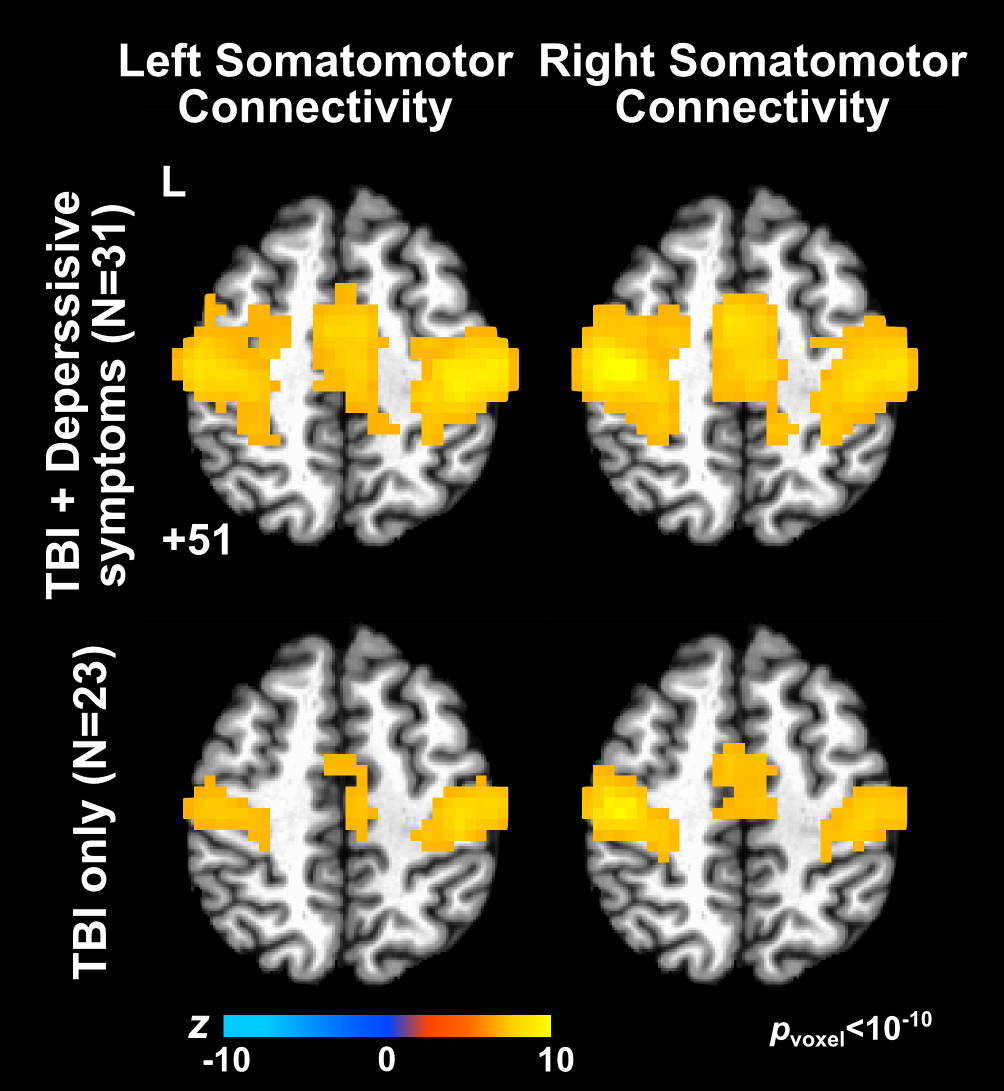

Supplement: Supplementary file 4 [file image_1.png]

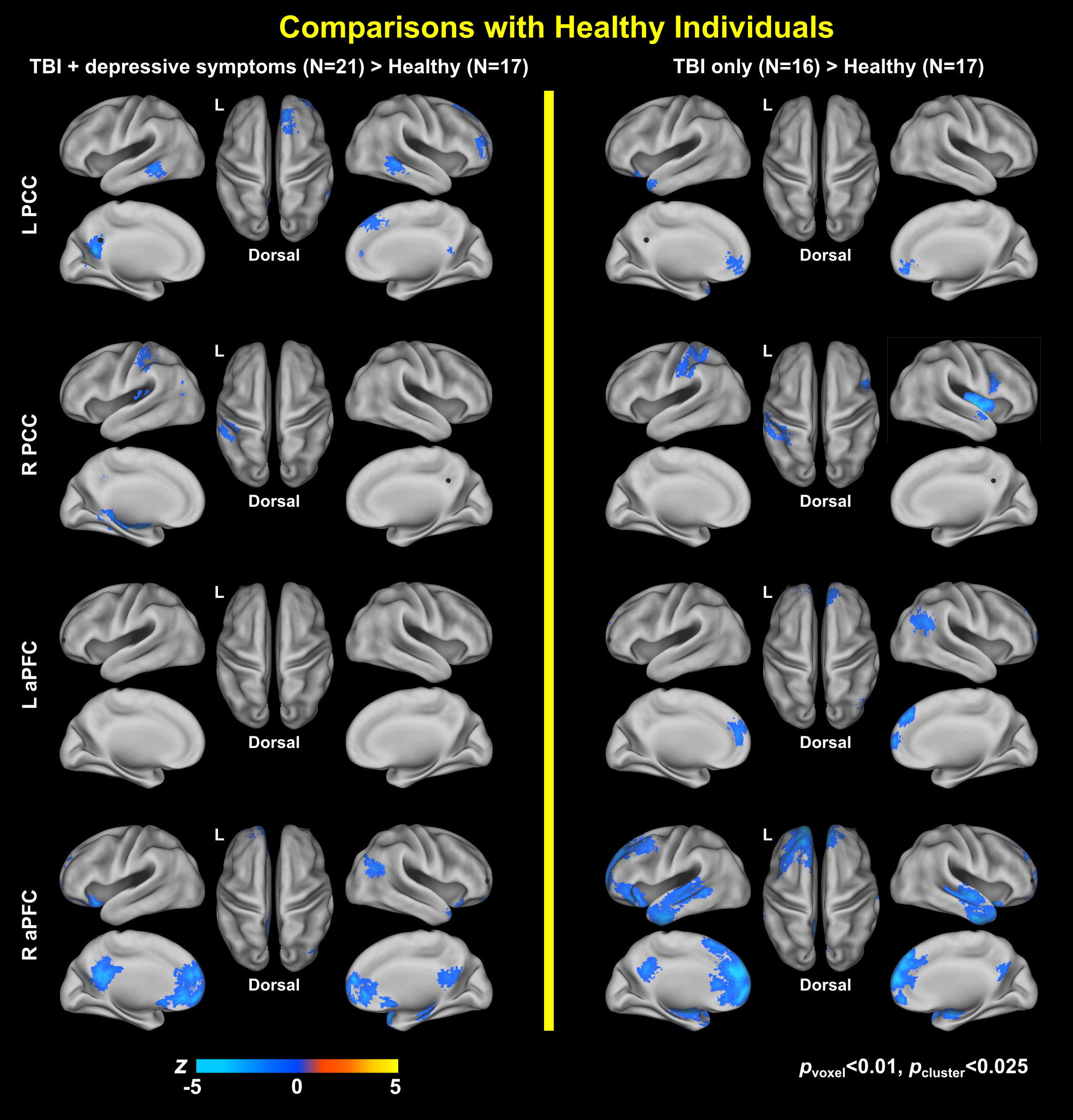

Supplement: Supplementary file 5 [file image_2.png]

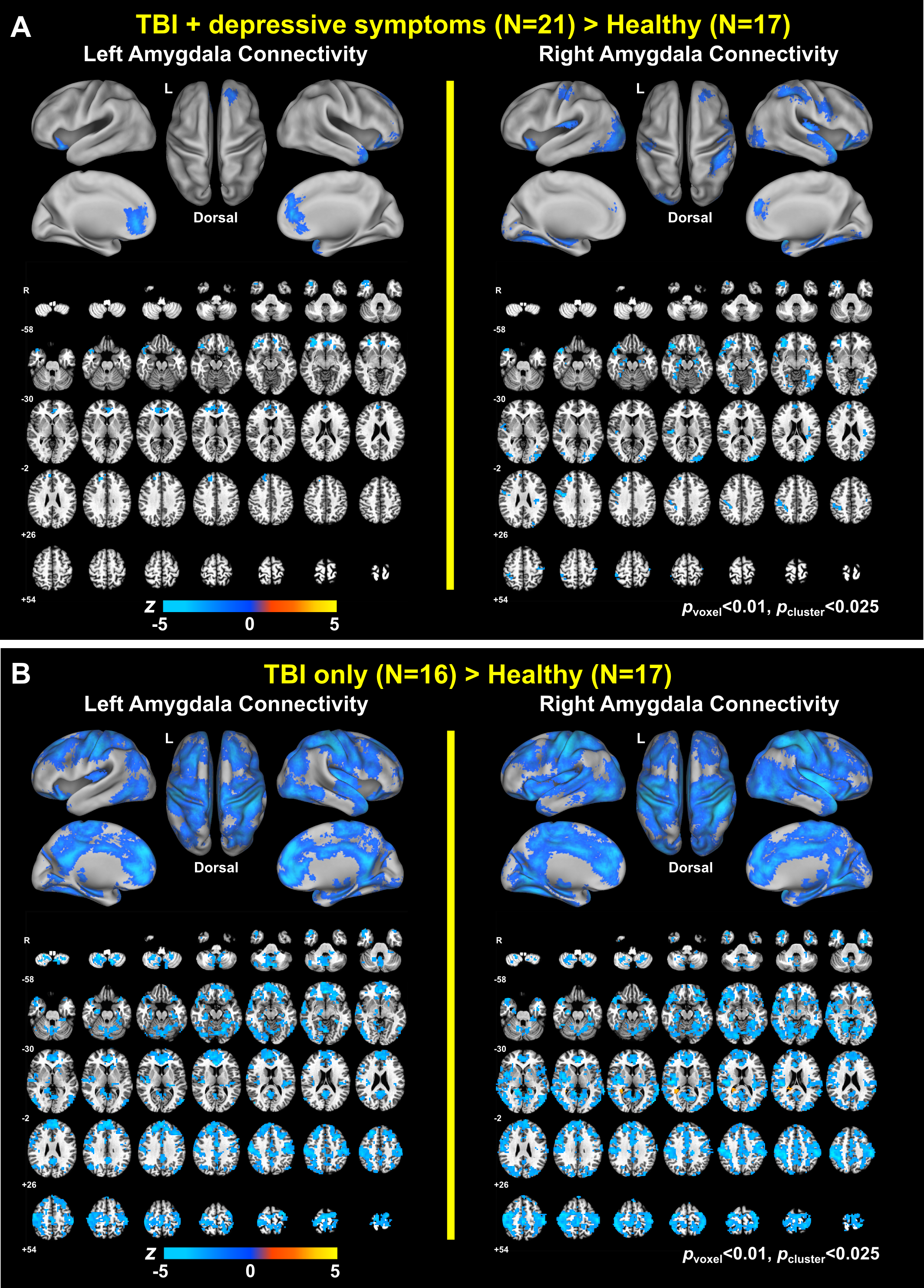

Supplement: Supplementary file 6 [file image_3.png]

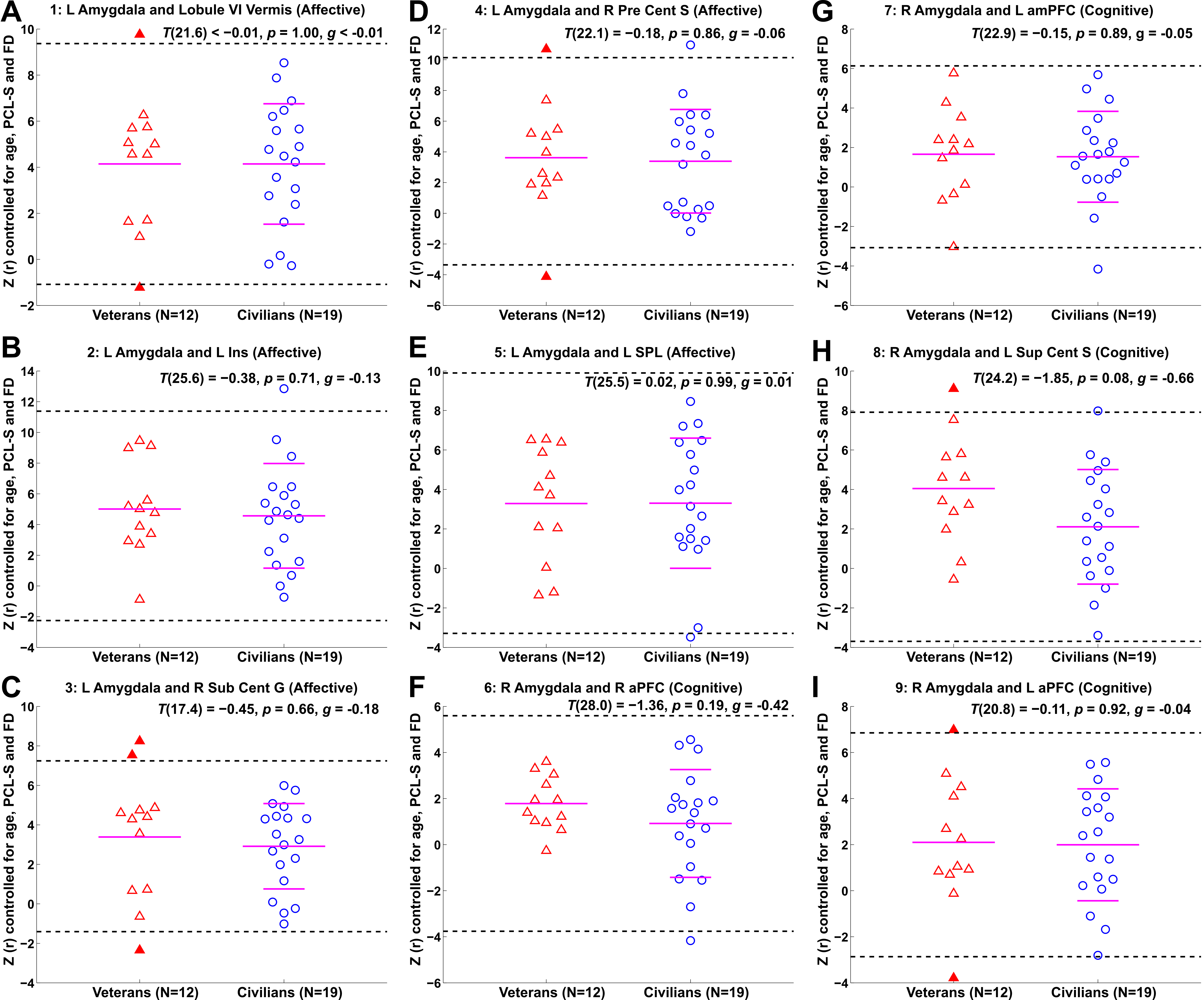

Supplement: Supplementary file 7 [file image_4.png]

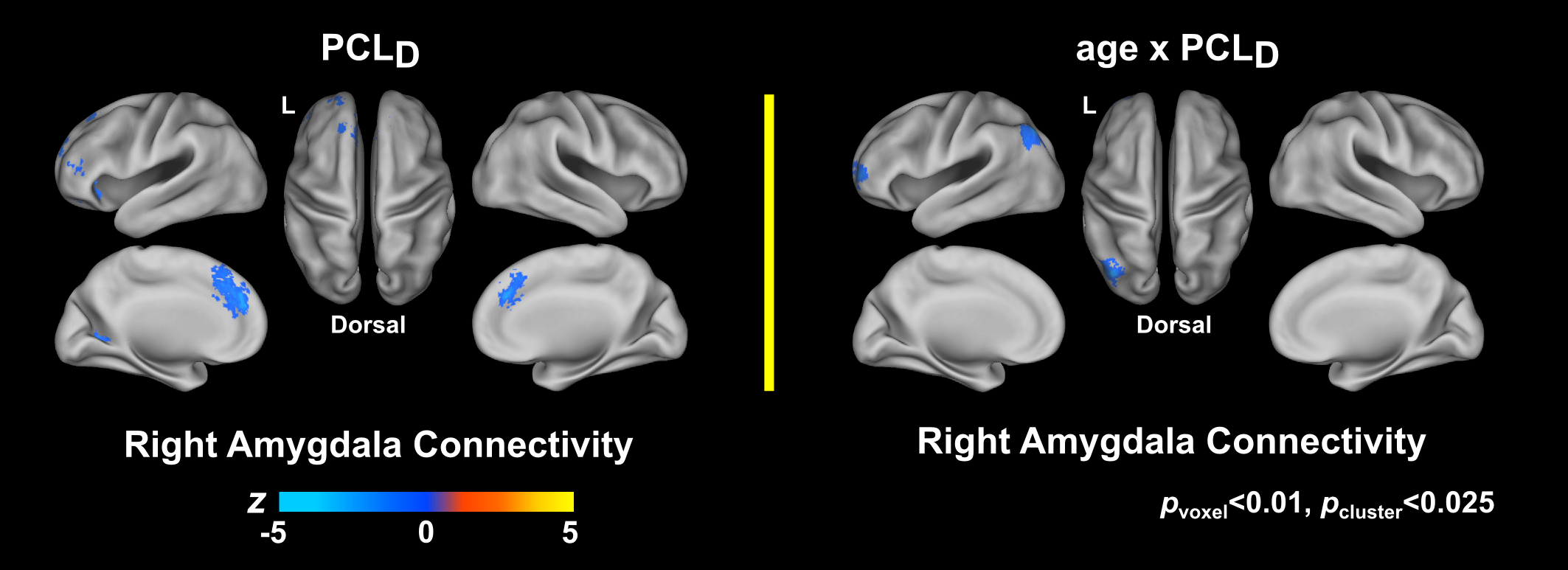

Supplement: Supplementary file 8 [file image_5.png]

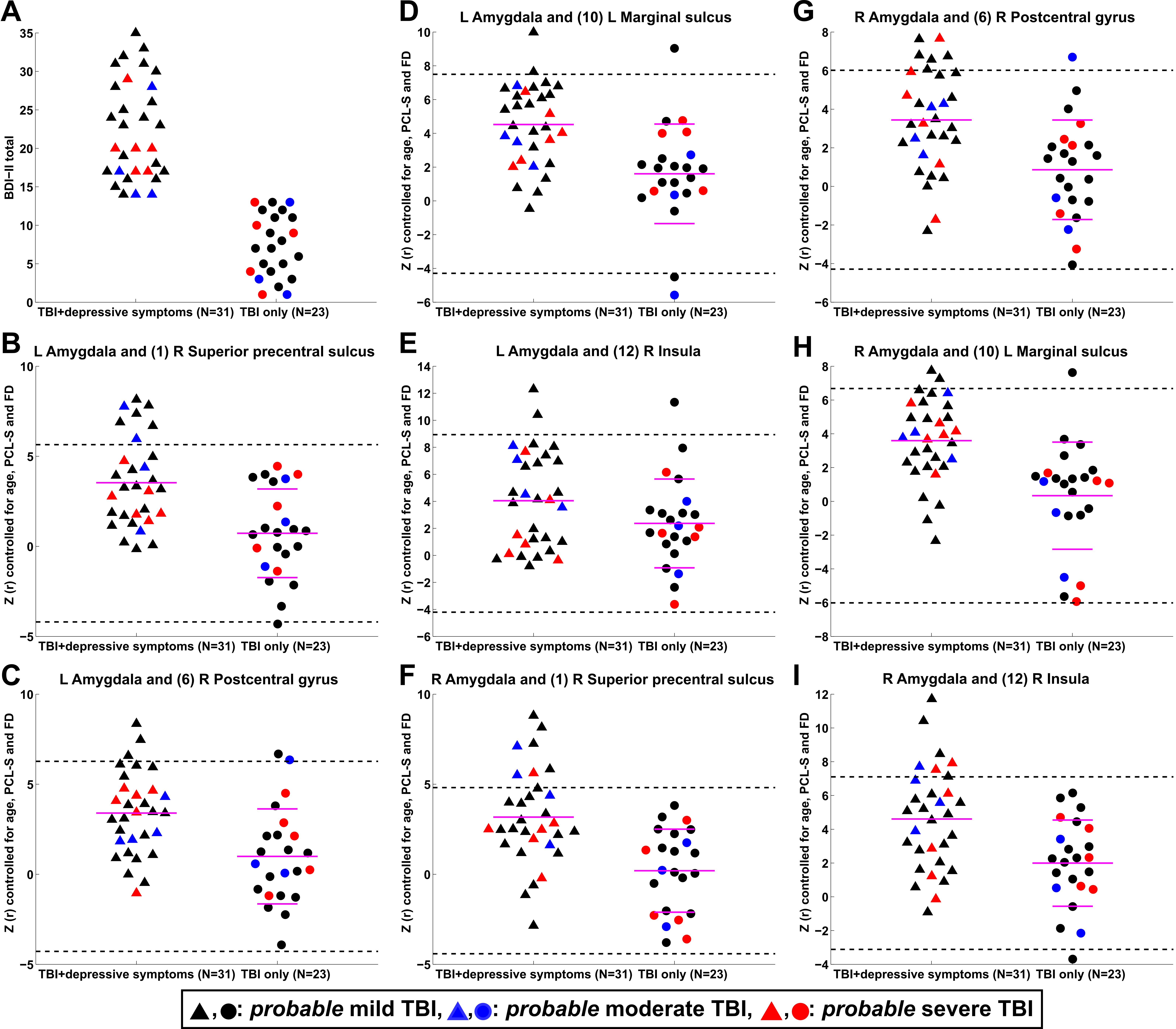

Supplement: Supplementary file 9 [file image_6.png]

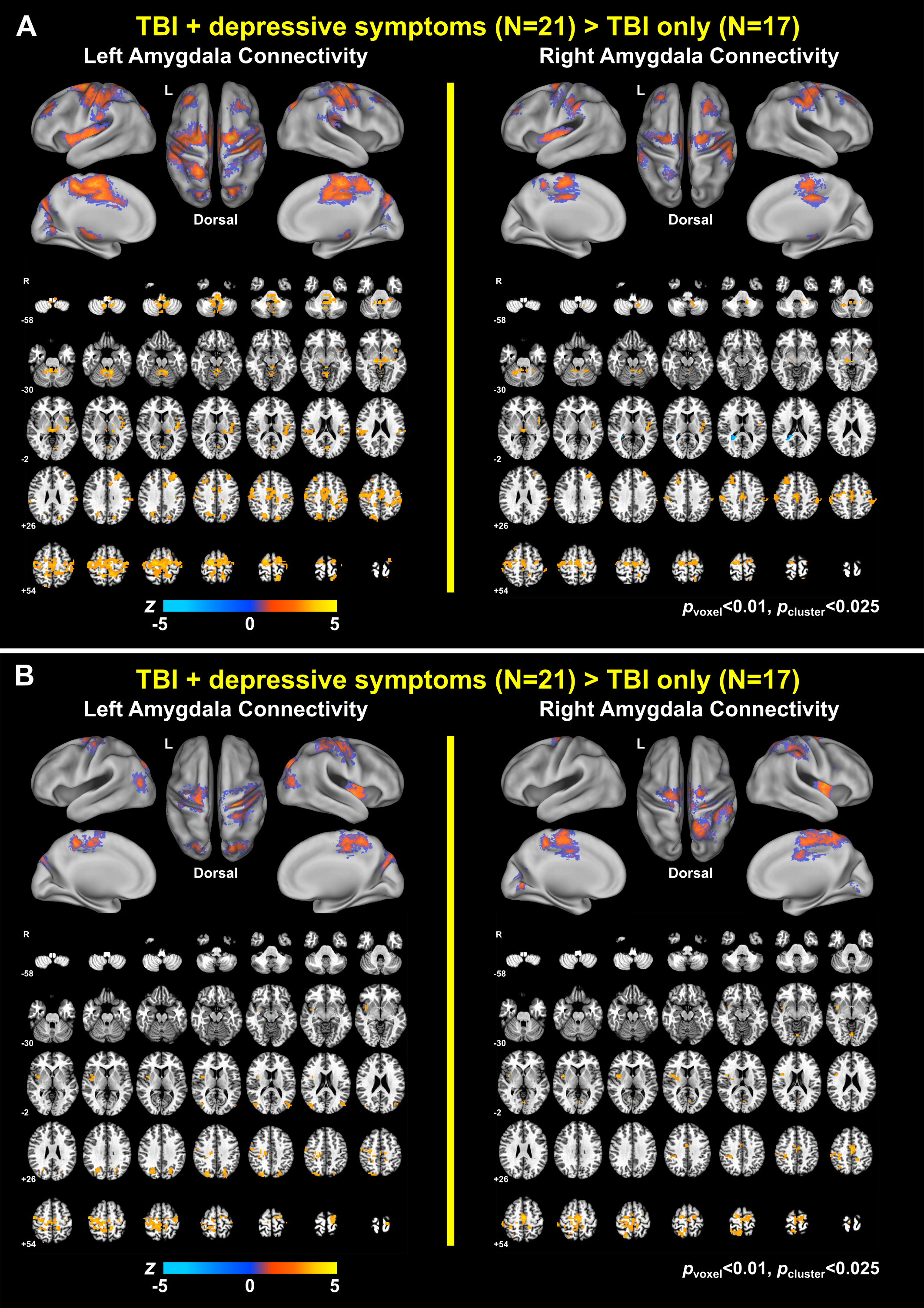

Supplement: Supplementary file 10 [file image_7.png]

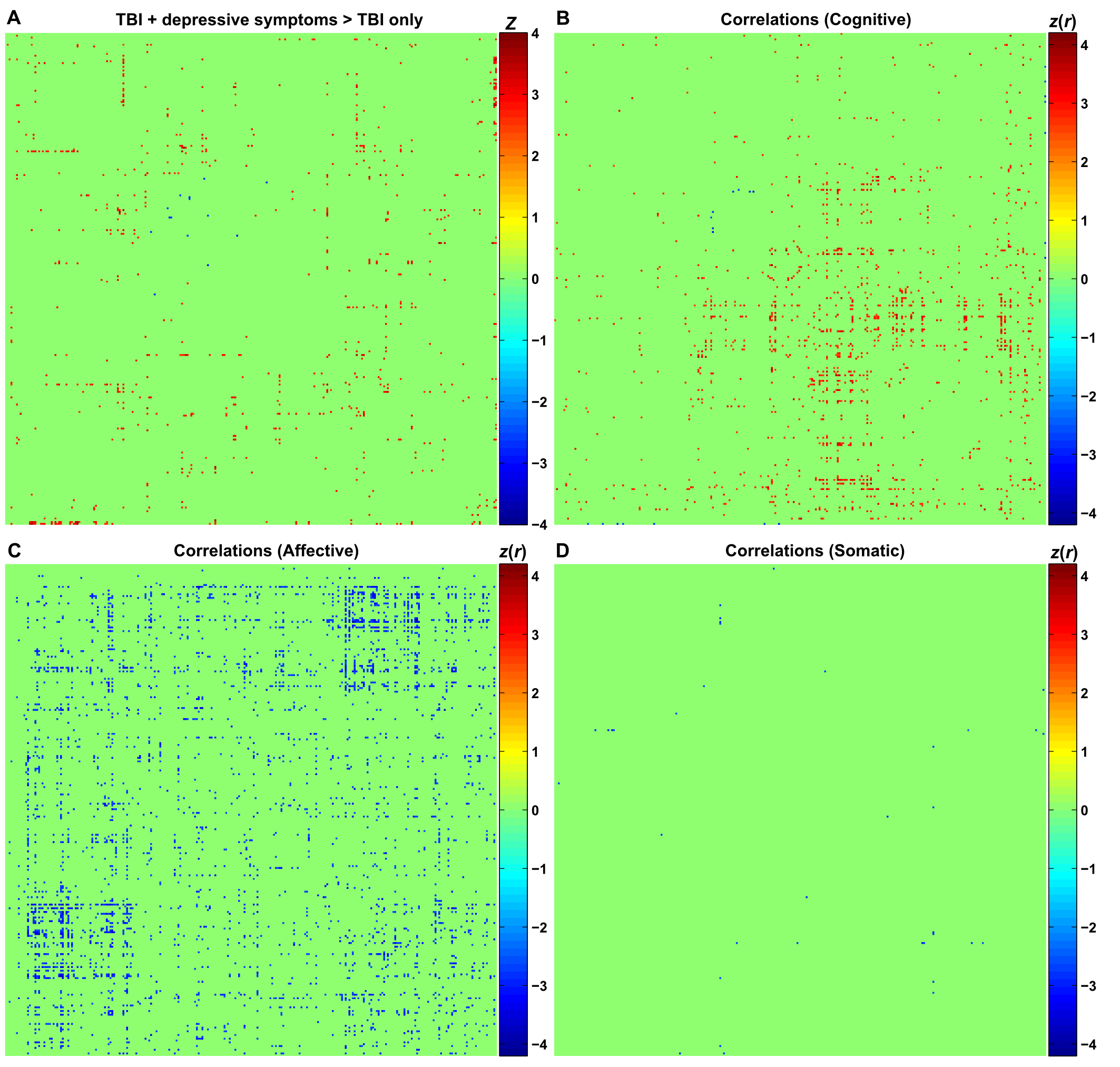

Supplement: Supplementary file 11 [file image_8.png]
